# Bat-Borne Viruses and Pandemic Risk: Could Europe Be an Emergence Hotspot?

**DOI:** 10.3390/v18050535

**Published:** 2026-05-02

**Authors:** Krzysztof Skowron, Justyna Bauza-Kaszewska, Anna Budzyńska, Natalia Wiktorczyk-Kapischke, Julia Czuba, Ewa Wałecka-Zacharska, Kacper Wnuk, Mariusz Zapadka, Krzysztof Kasprzyk, Katarzyna Grudlewska-Buda

**Affiliations:** 1 Department of Microbiology, Ludwik Rydygier Collegium Medicum in Bydgoszcz, Nicolaus Copernicus University in Toruń, 85-094 Bydgoszcz, Poland; skowron238@wp.pl (K.S.); a.budzynska@cm.umk.pl (A.B.); natalia12127@gmail.com (N.W.-K.); julia.czuba@cm.umk.pl (J.C.); 2 Department of Microbiology and Plant Ecology, Bydgoszcz University of Science and Technology, 85-796 Bydgoszcz, Poland; justyna.bauza-kaszewska@pbs.edu.pl; 3 Department of Food Hygiene and Consumer Health, Wrocław University of Environmental and Life Sciences, 50-375 Wroclaw, Poland; ewa.walecka@upwr.edu.pl; 4 Department of Theoretical Foundations of Biomedical Sciences and Medical Computer Science, Ludwik Rydygier Collegium Medium in Bydgoszcz, Nicolaus Copernicus University in Toruń, 85-067 Bydgoszcz, Poland; kacper.wnuk@cm.umk.pl; 5 Department of Inorganic and Analytical Chemistry, Ludwik Rydygier Collegium Medium in Bydgoszcz, Nicolaus Copernicus University in Toruń, 85-089 Bydgoszcz, Poland; mariusz.zapadka@cm.umk.pl; 6 Department of Vertebrate Zoology and Ecology, Nicolaus Copernicus University in Toruń, 87-100 Torun, Poland; kasprzyk@umk.pl

**Keywords:** bats, zoonotic viruses, transmission, bat-borne infections, Europe, pandemic risk

## Abstract

The recent SARS-CoV-2 pandemic—which had significant worldwide health, economic, and other effects—indicated the need to monitor zoonotic viruses with pandemic potential. The aim of this review is to assess bat-borne viruses as a potential pandemic risk, with a particular focus on Europe. The presence and activity of bats, as well as diseases emerging in humans in various regions of the world, point to their importance in the context of a possible outbreak of future epidemics. The rate of genetic change observed among viruses requires constant scrutiny on all continents, including Europe. Bats are a considerable source of many zoonotic viruses, including coronaviruses, filoviruses and paramyxoviruses. Among viruses associated with bats, RNA viruses are the dominant ones, characterized by high pathogenicity and often leading to interspecies transmission. The majority (about 80%) of RNA viruses were identified in bats from three families: *Vespertilionidae*, *Rhinolophidae* and *Pteropodidae*. Understanding how viruses are transmitted in the environment and the role of reservoir organisms and intermediate hosts is crucial to determining the level of epidemic risk. This review discuses viruses identified in bats globally, with a special focus on Europe, and evaluates their potential to cause epidemics.

## 1. Introduction

The Severe Acute Respiratory Syndrome Coronavirus 2 (SARS-CoV-2) global coronavirus disease 2019 (COVID-19) epidemic—which greatly affected the health, social and economic spheres—sparked debates about the original source. One hypothesis suggested the SARS-CoV-2 transferred from bats to humans, possibly via an indirect route involving another animal species. This theory was supported by studies confirming the high (96%) genomic similarity between SARS-CoV-2 and the RaTG13 virus found in *Rhinolophus* bats in China [1,2]. At the same time, the less than 90% similarity of the Receptor Binding Domain (RBD) between these viruses has raised doubts about the actual closeness of their phylogenetic relationship [3,4]. Bats have been considered a major reservoir of coronaviruses. Among the most dangerous and best-described are the following SARS-CoV virus responsible for the severe acute respiratory syndrome (SARS) epidemic that emerged in China in 2002, leading to 774 deaths, and the Middle East respiratory syndrome coronavirus (MERS-CoV), which caused the deaths of 803 people in 2012 in Saudi Arabia. The results of studies conducted at that time confirmed the possibility that bat-derived SARS-CoV and MERS-CoV were adopted by wild animals (civets, camels) and transmitted to humans. They also emphasized the key role of rapid virus evolution, through which coronavirus adaptation could occur in species that were not previously their reservoir [5,6,7].

**Figure 1:**
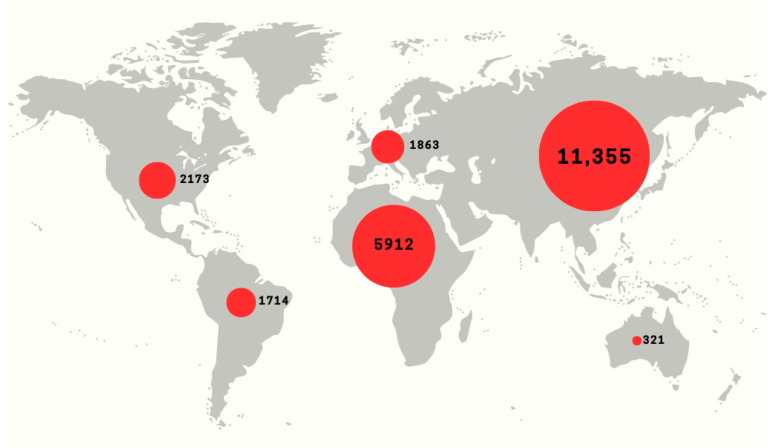
Global distribution of the number of currently identified bat-associated viruses, including figures for each continent (data for 1970–2026, from DBatVir database [14]).

The presence and activity of bats and the diseases appearing in humans in various regions of the world, indicates their importance in the context of possible outbreaks of further epidemics (Figure 1). Based on the results of studies conducted around the world, it can be concluded that the likelihood of such epidemics occurring in Asia or Africa is higher than in Europe [8,9,10]. However, the pace and direction of the genetic changes observed in viruses necessitate continued vigilance among epidemiologists in Europe. Data on viruses isolated from bats are limited across European countries, which highlights a significant gap in current knowledge. Since bats can harbor viruses associated with human and animal diseases, the risk of outbreaks of infections caused by them in any region of the world is an issue of high significance. In order to be better prepared to deal with future infections with pandemic potential, it is crucial to monitor animal–human interactions [11]. One of the measures advocated by scientists is to increase the collection and analysis of samples from wildlife, including bats. This may allow more reliable research that will inform the development of prevention strategies and estimation of the risk of mass spread of bat-viruses among humans [12,13]. Expanding surveillance, including for viruses with epidemic potential, is crucial for safeguarding public health.

## 2. Results

### 2.1. Bats—General Characteristics

Belonging to the order Chiroptera, bats are the only mammals able to fly. More than 1400 species inhabit almost the entire surface of the globe, except in locations characterized by extreme environmental conditions, such as deserts or the polar regions of Antarctica. They rely on a wide range of foods, often high in calories, such as nectar, fruits, insects, fish and small crustaceans, to obtain the energy necessary for long-distance flights. Energy loss minimizing is possible by taking advantage of the phenomenon of hibernation [15,16,17]. Their nocturnal lifestyle, specific roosting sites (caves, rock crevices, tree hollows, basements), ability to echolocate, and their feeding on blood—which is characteristic of some species—have influenced people’s pejorative perception of these animals and shaped their negative image in mass culture. Meanwhile, they play a relevant role in the global ecosystem by controlling populations of nocturnal insects that are harmful to crops or assisting in the pollination of plants and the spread of their seeds, which supports the restoration of forests. Nitrogen-rich bat droppings can also be used as a natural fertilizer [18,19].

The specific structure, characteristics and habits justify treating bats as an element of the ecosystem responsible for the spread of many etiological agents of human infections [20,21]. As shown by analyses of the available scientific data, bats host a much higher percentage of etiological agents responsible for zoonotic diseases than other orders of mammals [22]. In addition to viruses, bats have also been linked to the transmission of human and animal diseases caused by bacteria belonging to the genus: *Bartonella*, *Pasteurella*, *Leptospira*, *Campylobacter*, *Vibrio* and species of the *Enterobacteriaceae* family (genus: *Salmonella*, *Escherichia* and *Yersinia*), and the fungi *Histoplasma capsulatum* and *Pseudogymnoascus destructans* [18].

### 2.2. Bats as Hosts, Reservoirs and Vectors of Viruses—Biological, Physiological and Immunological Features

The uniqueness of bats in terms of their morphology, anatomy and social behavior makes them organisms with a special predisposition to spread viruses. Among the traits that determine these properties is their ability to fly, which obviously raises the risk of transmitting viruses over huge distances—up to 2000 km—and to places that could be potential sources of new intermediate or definitive hosts [16,23].

One of the key factors contributing to the significance of bats as reservoirs of pathogenic viruses is their ability to carry viruses without signs of infection. However, current knowledge of bat immunology is based on studies conducted in only a limited number of species, and several of the described traits may vary between taxa. Metabolic processes occurring during flight are one reason for the asymptomatic viral carriage often observed in bats. During flight, intense metabolic changes increase the bat’s body temperature reducing the rate of viral proliferation. Another hypothesis suggests that asymptomatic viral carriage in some bat species may be linked to modifications in the immune system, whose activity has been evolutionarily inhibited to reduce the risk of inducing inflammatory reactions in the body during each flight [16,24,25,26]. Considering the intensity of metabolic processes during flight, which is estimated to be up to 34 times higher compared to the basal metabolic rate, and the body temperature generated at the time—reaching 41 °C—these theories seem reasonable [16,17,18]. The adaptation of bats to control the replication of viruses in their own bodies and cope with the infections they cause is undoubtedly the result of a long evolutionary process undergone by these phylogenetically oldest mammals on Earth [23].

The asymptomatic reservoir status reported for some bat species is also associated with distinct immune responses described in the species studied so far. Among the mechanisms that determine innate immunity, a particular role is attributed to the constitutive nature of the expression of antiviral cytokines, mainly interferons (IFNs). The early IFN response helps in limiting the spread of the virus [24]. Viral infection in Egyptian fruit bats leads to the induction of type I IFN, while in pteropid bats, it triggers type III IFN [27]. IFN is also responsible for activating the expression of genes (ISGs) that control viral replication [24]. Irving et al. [16] have observed that the induction of some antiviral genes by IFN was specific only to bats and did not occur in other mammals. In addition to innate immune responses, the enhancement of bats’ defense mechanisms against viral infection is also provided by their high levels of heat shock proteins (HSPs), which enhance cellular tolerance to heat and oxidative stress, as well as an active autophagy mechanism that mediates the removal of pathogens from cells [16].

However, the effective response of the bat immune system to a viral infection may contribute to destructive changes in host cells as a result of the inflammatory response. Thus, the uniqueness of the response of bat organisms to the presence of a virus lies primarily in the balance between the activity of defense mechanisms and the effects of the system tasked with regulating the extent of potentially adverse changes associated with inflammation. One symptom of infection is the appearance of cytosolic DNA in cells, which triggers an acute immune response activated by the Stimulator of Interferon Genes (STING). In the bat species studied so far, however, this response appears to be suppressed by a point mutation identified within STING, specific only to these animals. This mutation attenuates the mechanisms responsible for immune responses, including IFN production [16,28].

External stressors, including natural environmental processes and increasingly destructive human activities, also contribute to virus transmission by bats. The resulting stress adversely affects the functioning of the bat’s immune system, increasing its susceptibility to infection by viruses from other individuals. Elevated seroprevalence in Hendra virus-carrying *Pteropus scapulatus* bats has been observed in individuals showing signs of nutritional stress resulting from, among other causes, the loss of their natural habitat. Significant energy costs associated with the immune response lead to increased susceptibility to viruses in populations under nutritional stress [29]. In turn, the need to search for new habitats often results in bats moving to areas of high population density and settling in the immediate vicinity of humans, increasing the risk of disease outbreaks [23].

The asymptomatic nature of viral infections in bats becomes more problematic due to their longevity, which is unusual for mammals of such mass and body size. Their lifespan of up to several decades (*Myotis brandtii*—up to 41 years) may contribute to the long persistence of the virus in their habitat. At the same time, an increasing number of serological studies suggest that the long-term persistence of viruses within bat colonies may be driven more by multiple reinfections rather than chronic infection, while recurring latent infections may also play a role [30,31]. Furthermore, the social dynamics of bat populations, based on the cohabitation of different species in large colonies that can exceed a million individuals, creates excellent conditions for virus transmission between individuals [15,16]. The exceptionally considerable number of bat species is undoubtedly related to the high diversity of viruses with zoonotic potential inhabiting their bodies [22,23]. Also, echolocation, accompanied by the generation of aerosols, may facilitate viral transmission, although not all bat species use this mechanism. Members of the Pteropodidae generally lack this ability, with some species of *Rousettus* being exceptions that echolocate using tongue clicks. Like morphology, echolocation is a flexible trait shaped largely by ecological demands [32,33].

### 2.3. The Genetic Aspects of Bat-Borne Viruses

The risk associated with pathogenic viruses in bat organisms increases with their ability to adapt to new hosts. In order to do this, however, viruses must overcome barriers existing in all living organisms which impede their effective execution of the typical stages of infection. The mechanisms enabling successful infection of new hosts are often a part of an evolutionary process to expand the range of potential hosts. Such an evolutionary process has been observed in a number of viruses that use, in order to enter cells, highly conserved molecules that function as receptors, which are very similar genetically in different animal species. In the case of betacoronaviruses, they bind to various cell surface proteases at the cell entry stage, which are almost genetically identical in bats, camels and civets, as well as humans. Increased compatibility with receptors found in different bat species may result from rapid single point mutations, as confirmed in MERS-CoV [25]. A high mutation rate is one of the factors responsible for the genetic diversity of RNA viruses, while in the case of SARS and MERS, the evolutionary “engine” is rather a recombination. It usually results in multiple simultaneous mutations, shaping new viral traits, including altered host ranges and an increased ability to spread in a new host [34]. Scientists have suggested that the human SARS-CoV virus arose precisely from the recombination of multiple separate but related viruses detected in bats. The full SARS-CoV genome has been sequenced from genetic material obtained from all of these strains of bat-associated viruses [25]. Although most recombinations involve homologous sequences between related viruses, results are also available confirming the possibility of inter-family recombination between coronaviruses and reoviruses [35].

### 2.4. Spillover Routes of Bat-Associated Viruses

**Figure 2:**
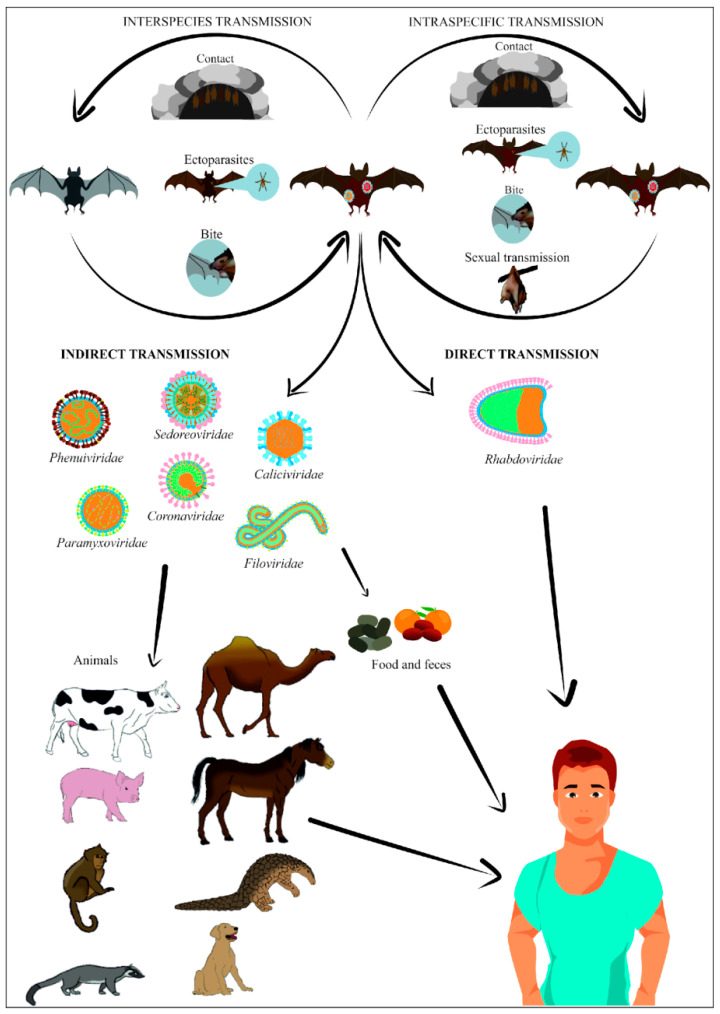
Possible routes of transmission for bat-borne viruses. Direct transmission includes: direct contact between bats and humans (rare, applies to lyssaviruses), including through skin breaks (bites or scratches) or the consumption of infected bat meat (e.g., Ebola virus). Indirect transmission routes involve intermediate hosts (primarily domestic and farm animals)—infection of intermediate hosts can occur through the consumption of food that has been partially digested and then regurgitated by bats; indirect contact with bat feces (including consumption of raw and unwashed fruit or contaminated palm juice, likely contaminated with bat saliva, urine, or feces).

Viruses can spread in the environment through various transmission routes: direct and indirect (Figure 2). Infections due to direct bat–human contact are rather sporadic and mainly involve lysaviruses responsible for rabies. The direct route of virus transmission involves damage to the skin caused by a bat bite or scratch [36]. Direct human infection also occurs through infected bat meat consumption [37]. It is believed that contact with bat meat, which was a putative reservoir for the Ebola virus, may have been the cause of 186 deaths in Africa in 2007 [38]. Also capturing and butchering bats poses a high transmission risk. Much more frequent human infections by bat-derived viruses occur through intermediate hosts, among which pets and livestock pose the greatest threat. Infected pigs and horses have been the cause of morbidity and deaths in humans caused by Nipah Virus (NiV) and Hendra (HeV) viruses, respectively [17]. It is important to note that HeV infection has caused only seven confirmed human cases to date, including four fatalities, indicating that human infections are rare and limited to sporadic spillover events [39]. In contrast, NiV infection has been responsible for over several hundred human cases worldwide, with recurrent outbreaks reported particularly in South and Southeast Asia [40]. The intermediate hosts in the SARS-CoV outbreak, on the other hand, were civets and possibly raccoons [17]. Infection of intermediate hosts can occur as a result of the ingestion of food that has previously been partially digested and then spat out by bats due to their limitations in absorbing foods (ingested food would burden the bats’ bodies, disturbing aerodynamics of their flight). In order to obtain and store the energy necessary for life processes, they use the high-calorie and nutrient-rich juices produced after chewing the soft tissues of the fruits. Unnecessary and heavy residues of chewed food are spat out by the bats, thus introducing potentially virus-contaminated material into the environment [18]. In addition to saliva, urine and their other secretions can also be a source of human and animal infection. Indirect contact with bat droppings has been suggested as a route of Marburg virus transmission in four Ugandan miners working at *Rousettus aegyptiacus* nesting sites [41,42]. Similar concerns have been raised about the potential risk of group C beta-coronavirus (β) infection of miners extracting bat guano after these viruses were isolated from the feces and urine of these animals.

On the other hand, an indirect route of transmission could involve consuming food that has come into contact with secretions from infected bats (Figure 2). In the case of NiV outbreaks in Bangladesh and India, most human infections have occurred through the consumption of raw and unwashed fruits or contaminated palm sap, probably contaminated with bat saliva, urine, or feces. Fruit bats, primarily belonging to the genus *Pteropus*, are often implicated in these outbreaks [43]. Researchers have suggested that the HeV, found in bats’ urine, could contribute to the disease in horses, which massively infected humans working in their vicinity [44]. The diversity of the transmission routes of viruses transmitted by bats to intermediate hosts or directly to humans determines their importance in the epidemiology of viral diseases.

### 2.5. The Most Dangerous Viruses Carried by Bats

Bats are a considerable source of many zoonotic viruses, a significant number of which—especially those belonging to the coronaviruses, filoviruses and paramyxoviruses—have a significant impact on global public health. At the same time, rodents represent the second largest mammalian order and are also recognized as major reservoirs of zoonotic pathogens, including hantaviruses and arenaviruses. They are estimated to host approximately three times more unique pathogens with zoonotic potential than bats. The highest diversity of rodent host species is observed in northern regions of North America and Europe, as well as in Brazil [45,46,47]. However, it remains unclear to what extent the higher diversity of rodent host species observed in these regions reflects true ecological differences versus increased sampling effort and reporting intensity in well-studied regions. Compared to other animal reservoirs of viruses, bats are the source of the most virulent ones, even when compared to birds. Importantly, no single animal reservoir, including bats, is uniquely associated with a high human mortality burden [48].

Due to their exceptional species diversity, wide geographic distribution, and ability to host a large number of RNA viruses, bats are considered one of the most important wildlife reservoirs of emerging zoonotic viruses. Advances in molecular techniques have significantly expanded the ability to detect novel viruses in bats and assess their potential role as reservoirs of emerging pathogens. In particular, metagenomic next-generation sequencing (mNGS), deep sequencing, and massively parallel sequencing enable the simultaneous analysis of genetic material from all organisms present in a sample, allowing the identification of viruses with very low similarity to previously known viral genomes. Using these approaches, numerous previously unknown viruses have been detected in bat populations. Although much of this research has been conducted in regions considered hotspots for emerging infectious diseases, similar discoveries in European bat populations demonstrate that novel bat-associated viruses are not limited to high-risk regions [49,50,51,52,53,54]. Deep sequencing of microchiropteran bats in Germany identified a new banyangvirus [55]. In Spain, a new lyssavirus was detected in the Iberian bat, and three complete potential novel coronavirus genomes were recovered, while the Kotalahti bat lyssavirus was later identified in Finland in Brandt’s bat [56,57,58].

Among viruses associated with bats, RNA viruses are the dominant ones, characterized by high pathogenicity and often leading to interspecies transmission. The majority (about 80%) of RNA viruses was identified in the bats of three families: *Vespertilionidae, Rhinolophidae* and *Pteropodidae* [59].

**Figure 3:**
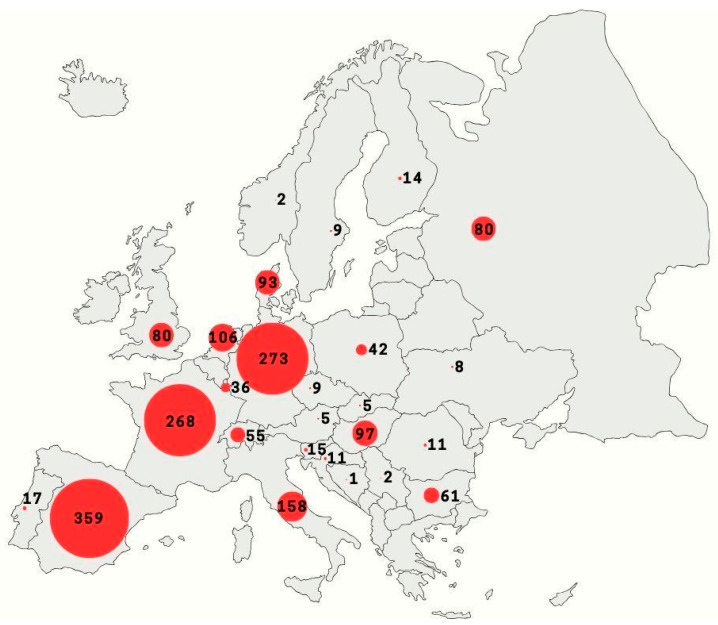
Distribution of the number of bat-associated viruses currently identified in Europe, along with data for each European country (where available) (data for 1970–2026, from DBatVir database [14]).

Based on information from DBatVir [14], a global database that collects information on viruses carried by bats, the oldest bat tissue samples in which human pathogenic viruses have been confirmed date back to 1968. The virus in question was European bat 1 lyssavirus isolated in Germany. The growing number of analyzed samples and improvements in virus identification methods have undoubtedly allowed the detection of many viruses. Currently, DBatVir base covers 1456 (November 2025) bat-associated viruses in Europe (Figure 3) and since 2003 *Rhabdoviridae* have ceased to be the only family of viruses detected in bats. This date coincides with the first coronavirus outbreak caused by SARS-CoV-1 in China, and the representatives of *Coronaviridae* that have been appearing more and more frequently alongside *Rhabdoviridae* and other families in bat-borne virus statistics. Beginning in 2020, all entries included in the databaset are exclusive to *Coronaviridae*.

#### 2.5.1. Family *Coronaviridae*

A few years ago, scientists considered the *Rhabdoviridae*, including the rabies virus, the most important family of zoonotic viruses associated with bats. Currently, the coronaviruses, with SARS and MERS at the top, are generally mentioned first. The *Coronaviridae* family includes the largest known single-stranded positive-sense RNA viruses, which are enveloped particles about 118–140 nm in diameter and genomes of approximately 26–32 kb—the largest genome size currently known among RNA viruses [60]. Within the family are four subfamilies, Coronavirinae or Orthocoronavirinae, Letovirinae, and Pitovirinae. The Coronavirinae includes four genera: the mammal-infecting Alphacoronavirus and Betacoronavirus, and the bird- and mammal-threatening Deltacoronavirus and Gammacoronavirus [61,62,63]. The SARS-CoV, MERS-CoV, and SARS-CoV-2 belong to Betacoronavirus (beta-CoV), with SARS being within the Sarbecovirus subgenus and MERS being within the Merbecovirus subgenus [64,65,66].

The COVID-19 outbreak, caused by the SARS-CoV-2 has resulted in 779,177,817 confirmed cases of illness and 7,113,407 deaths worldwide [67]. COVID-19 sparked a surge of interest in coronaviruses and the potential source of their emergence further or closer to humans. Coronaviruses are capable of infecting a wide range of vertebrate hosts, including mammals and birds. Natural infections have been documented in numerous species such as bats, camels, civets, pangolins, rodents, cats, dogs, and minks [68,69,70,71,72,73]. Early epidemiological investigations during the SARS outbreak in 2002–2003 suggested that civets acted as intermediate hosts transmitting the virus to humans, whereas bats of the genus *Rhinolophus* were later identified as the most likely natural reservoir of SARS-related coronaviruses.

After the COVID-19 pandemic in China in 2019, its origins were initially sought in human–animal interactions, although there were several hypotheses regarding the origin of SARS-CoV-2. The assumption that the virus originated in the Wuhan Institute of Virology laboratory and was introduced into the environment by an infected worker seemed highly unlikely. However, discussions related to this are still ongoing mainly because the Chinese government, which denies the SARS-CoV-2 laboratory origin, prevents adequate efforts to find evidence of its absence [74]. A more likely hypothesis was that animals were the source of the pandemic’s onset, indicating the SARS-CoV-2 transmission both directly from bats to humans and with the involvement of an intermediate host [75]. Vulnerability to natural or experimental infection with the virus has already been demonstrated for 42 animal species (Last update: 25 March 2025), with the highest number of SARS-CoV-2 events reported in mink, cats and dogs [76]. The Huanan market in Wuhan sells a variety of animal species, such as badgers, palm civets, raccoons and minks. Worobey et al. [77] pointed to the sale of animals that are likely intermediate hosts of SARS-CoV-2. The results presented by Liu et al. [78] showed positive test results for environmental samples from the market but not for animal samples. Scientists, however, quickly criticized the quality and consistency of the tests conducted [78]. Since no virological samples were taken before the slaughter and decontamination of the Huanan market on 1 January 2020, and origin tracing of the sold mammals is impossible, it is difficult to pinpoint the primary reservoir of SARS-CoV-2 and its intermediate host. The viruses, with confirmed close association with SARS-CoV-2 by metagenomic studies and phylogenetic analyses, have been detected in free-living horseshoes (*Rhinolophus*) from several Asian countries [79]. Direct transmission of SARS-CoV-2 from horseshoe bats to humans is considered unlikely. Scientists indicate pangolins as potential intermediate hosts since they are often sold illegally on the Chinese market for food, and their scales are used in traditional medicine. However, the genome of the viruses detected in these animals is too different from that of SARS-CoV-2, negating this hypothesis [74,80]. The genetic proximity and the presence of virulence factors similar to SARS-CoV-2 found in viruses isolated from wildlife prove that the pandemic threat has not lost its relevance. Therefore, it is important to counter the spread of pathogens through increased inspections of wet markets where legal and illegal wildlife trade occurs. In addition, workers on farms breeding animals should be surveyed, and susceptible populations, such as mink, should be monitored [81]. Preventive strategies may also include vaccination programs targeting susceptible animal species that can act as intermediate hosts, such as pigs, horses, or minks [82,83]. Studies by Bashor et al. [68] have shown the potential in cats for new virus variants to emerge more rapidly than in humans, confirming the need for the continuous monitoring of animals, particularly domestic animals, and the full genome sequencing of SARS-CoV-2. The aforementioned preventive measures are elements of the One Health approach, defined as “an integrated, unifying approach that aims to sustainably balance and optimize the health of people, animals and ecosystems” [84,85].

Detected in 2012 in Saudi Arabia, MERS-CoV, which causes respiratory illnesses with high mortality rates, has appeared in many countries worldwide, including Europe. Coronaviruses similar to MERS-CoV have been detected in bats of the genera *Pipistrellus*, *Nycteris* and *Neoromica*. At the same time, researchers have identified many MERS-CoV lines in camels, which may suggest that they were intermediate hosts for the MERS-CoV virus, originally from bats [17,44].

European insectivorous bats harbor diverse coronaviruses. Table S1 presents cases of coronavirus isolation from bats in Europe from 1970 to 2026, based on data from the DBatVir database, providing a comprehensive overview of the occurrences discussed in this section. For instance, a survey in northern Germany detected ~10% prevalence of alphacoronaviruses across several bat species. Although these bat coronaviruses form clades related to known human coronaviruses none has been reported to infect humans in Europe so far [86].

#### 2.5.2. Family *Rhabdoviridae*

The family *Rhabdoviridae* includes negative-strand RNA viruses containing genetic material of 10–16 kb. Among them are a number of plant, animal and human pathogens [16,87]. Within the *Lyssavirus* genus, which is part of this family, is the rabies virus, which, by attacking the central nervous system, usually leads to the death of the infected person. It is transmitted to humans mainly by carnivores (dogs, foxes, cats), but its reservoir hosts are probably also bats. Bats are important reservoirs of a diverse range of lyssaviruses, not limited to the Rabies virus. In particular, several distinct bat-associated lyssaviruses have been identified in Europe, including European bat lyssavirus 1 and European bat lyssavirus 2, which can cause rabies-like disease in humans and other mammals. Many bat species carry lyssaviruses without apparent clinical disease manifestation; however, symptomatic infections have also been reported [88]. Bats are recognized reservoirs of a diverse range of lyssaviruses, including European bat lyssavirus 1 (EBLV-1) and European bat lyssavirus 2 (EBLV-2) identified in Europe. Rabies in bats is etiologically and epidemiologically distinct from “classical” dog-mediated rabies, despite both being caused by viruses belonging to the genus Lyssavirus. Classical rabies is primarily associated with Rabies virus (RABV) maintained in domestic dog populations and remains the dominant source of human rabies cases globally, particularly in regions where canine vaccination coverage is insufficient [89].

Differences in the pathogenesis of human rabies following exposure to bats versus dogs are thought to be associated with the nature and depth of tissue injury at the site of bite. Bat-acquired rabies, which may be underdiagnosed, is often associated with superficial skin exposure, allowing the virus access primarily to peripheral endings of somatic sensory neurons, whereas dog bites typically involve deeper tissue penetration and more direct access to peripheral nerves within muscle. It has been suggested that bat-associated lyssaviruses may be better adapted to initiate infection at superficial cutaneous levels, which may contribute to differences in clinical recognition compared with classical dog-mediated rabies [90].

Despite the relatively low likelihood of direct contact between bats and humans and the low risk of injury that would allow the virus to enter the human body, the consequences of these incidental events are particularly serious due to the lack of an effective drug [91,92,93].

Bats carrying lysaviruses inhabit various geographic regions worldwide—Australia, Asia, Europe (Table S2) and the Americas. In South and Central America blood-eating vampire bats, belonging to the genera *Desmodus*, *Diphylla* and *Diaemus*, among others, are responsible for more cases of rabies in humans than dogs [17,44]. Although surveillance varies across Europe, evidence indicates that bat rabies is widespread on the continent [94]. Over recent decades, more than 1100 cases of bat rabies have been reported in Europe (especially in Denmark, Germany, Netherlands, France and Poland) [95]. At least five human deaths due to European bat lyssaviruses (EBLV-1 and EBLV-2) have occurred in Europe (1977–2002) [96].

#### 2.5.3. Family *Paramyxoviridae*

The most well-known and epidemiologically relevant genus of viruses within the *Paramyxoviridae* family is undoubtedly *Henipavirus*, which includes NiV and HeV viruses characterized by a high pathogenicity towards humans [97]. These negative single-stranded RNA viruses, ranging in size from 40 to 1900 nm, are responsible for zoonotic diseases deemed a priority by the WHO.

Researchers have reported the highest number of cases of animal and human illness and death caused by zoonotic paramyxoviruses in Australasia [44]. HeV was first described in Australia in 1994, where a number of deaths, resulting from acute respiratory illness in horses and encephalitis in humans, occurred [98,99]. Then, virus-neutralizing antibodies were identified in a number of bat species of the genus *Pteropus*: black flying fox (*P. alecto*), gray-headed flying fox (*P. poliocephalus*), ocular flying fox (*P. conspicillatus*) and little red flying fox (*P. scapulatus*) [100].

On the other hand, NiV first appeared in breeding pig populations in Malaysia, followed by Singapore, India and Bangladesh in the late 20th/early 21st century [43,101]. The average mortality rate for NiV ranged from 40 to 90% [102,103]. Scientists have searched for the NiV virus reservoir among bats, detecting virus-neutralizing antibodies in the large flying fox (*P. vampyrus*), the small flying fox (*P. hypomelanus*), the cave nectar bat (*Eonycteris spelaea*), the smaller short-eared fruit bat (*Cynopterus brachyotis*) and the smaller Asian yellow bat (*Scotophilus kuhlii*). The Indian flying fox (*P. medius*), meanwhile, has been linked to strains of NiV virus that dominated human outbreaks in India and Bangladesh in 2001 [17,104]. Both the Malaysian and Bangladeshi/Indian outbreaks of NiV infection were caused by distinct genetic lineages NiV-MY (Malaysia/Singapore lineage) and NiV-BD (Bangladesh/India lineage), which differ in their epidemiological characteristics. NiV-MY was responsible for the 1998–1999 outbreak in Malaysia and Singapore. Transmission occurred primarily through spillover from fruit bats to domestic pigs, which acted as an intermediate amplifying host, followed by pig-to-human transmission. Human-to-human transmission was rare or absent, and cases were largely associated with occupational exposure in pig farming and slaughterhouse settings [105]. NiV-BD (Bangladesh/India lineage) is associated with repeated outbreaks in Bangladesh and India. Transmission is primarily linked to spillover from fruit bats to humans, with ingestion of contaminated raw date palm sap being the best-established route in Bangladesh. In India, the exact spillover route has not been consistently established, although human-to-human transmission has been documented, particularly in healthcare settings [106].

The third of the bat-borne zoonotic viruses in the *Paramyxoviridae* family is Menangle virus, identified in Australia in 1997 as the etiological agent responsible for reproductive disorders in pigs. The symptoms of infection that occurred in humans were flu-like in nature and ended with the patients recovering. Asymptomatic carriers of the virus were flying bats: *P. poliocephalus*, *P. alecto* and *P. conspicillatus* [107,108,109].

Notably, henipaviruses (NiV and HeV) do not circulate in Europe: their natural reservoir are fruit bats of genus *Pteropus*, which occur only in Asia/Oceania. Thus far, NiV virus outbreaks have been restricted to Asia [110]. This indicates current risk in Europe is low, though travel/import routes should be considered. In addition, serological and molecular evidence suggests that apathogenic henipaviruses are circulating in bats and can spill over to livestock and humans in Africa, often without reported clinical disease. However, their zoonotic potential, as well as clinical significance, remains unclear [111,112].

#### 2.5.4. Family *Filoviridae*

The *Filoviridae* family contains two very dangerous genera for humans—Marburgvirus (MARV) and Ebolavirus (EBOV)—which are responsible for severe hemorrhagic fevers [113,114]. These single-stranded, negative-sense RNA viruses are detected periodically in different regions of Africa, and the diseases caused by them have a very high mortality rate [115].

Researchers have identified the Egyptian fruit bat *Rousettus aegyptiacus* as the likely natural reservoir host of MARV—the source of infection for miners working in the caves they inhabit in Uganda [116,117]. Although viral loads detected in rectal swabs were relatively low, they may still represent a potential route of viral shedding and facilitate transmission between bats, as well as spillover to humans and non-human primates. This suggests that excretory products may play an important role in MARV transmission dynamics within bat colonies and potentially across species barriers, without requiring direct physical contact such as biting or handling of infected animals. Furthermore, disturbance of roosting bats by human entry can trigger mass takeoff, during which bats frequently excrete urine and feces in flight or immediately upon departure [116].

EBOV antibodies, on the other hand, have been detected in various bat species in Africa and in Philippine *Rousettus amplexicaudatus* bats [118]. Scientists have found the Ebola virus-specific immunoglobulin G in the serum of three species of African fruit bats: hammer-headed fruit bat (*Hypsignathus monstrosus*), Franquet’s epauletted fruit bat (*Epomops franqueti*) and little collared fruit bat (*Myonycteris torquata*). Consumption of fruit bat meat was considered the root cause of the 2007 Ebola outbreak in the Democratic Republic of Congo (DRC), affecting more than 260 people, 186 of whom died [38,44]. In contrast, the straw-colored fruit bat (*Eidolon helvum*) is regarded as a likely, but not definitively confirmed, reservoir of EBOV. Evidence supporting its role includes serological data indicating widespread exposure across African populations and occasional detection of filovirus RNA [117,119]. *E. helvum* is considered a strong candidate reservoir or part of a broader reservoir system for EBOV, its exact role in virus maintenance and transmission in nature is still not fully resolved.

The only known European filovirus is Lloviu virus (LLOV), which was first found in Schreibers’s bats in Spain (2002) and later in Hungary (2016) [120]. Infectious LLOV has been isolated from bat blood and can infect human cells in vitro, suggesting zoonotic potential similar to Ebola virus [120].

#### 2.5.5. Family *Hantaviridae*

Infections caused by hantaviruses, belonging to the Bunyavirales order, have been reported in Europe, Asia, Africa and the Americas. Old World hantaviruses cause hemorrhagic fever with renal syndrome (HFRS) in humans, while in the Americas they cause hantavirus cardiopulmonary syndrome (HCPS). Although their transmission was initially attributed solely to rodents, researchers have detected bat-associated hantaviruses in Asia, Africa and Europe [121,122]. Among the recently described new bat-hantaviruses found in Africa are Mouyassue virus (MOYV), Magboi virus (MGBV) and Makokou virus (MAKV). In Asia, most hantaviruses have been identified in bats from China—Huangpi virus (HUPV), Longquan loanvirus (LQUV), Laibin mobatvirus (LBV) [123,124,125].

Hantaviruses are important zoonoses in Europe (e.g., Puumala virus causes HFRS). A novel bat-borne hantavirus (Brno virus) was recently identified in common noctule bats (*Nyctalus noctula*) in Central Europe, suggesting that European bats may also harbor hantaviruses [126].

#### 2.5.6. Family *Phenuiviridae*

The best-known virus belonging to the *Phenuiviridae* family is the highly pathogenic Rift Valley Fever Virus (RVFV), detected in Guinea in *Micropteropus pusillus* and *Hipposideros cafer* bats [123]. In humans the RVFV contributes to both self-limiting disease and severe cases of encephalitis associated with retinitis, hepatic necrosis, and fatal hemorrhagic fever. Although the virus is primarily transmitted by mosquitoes, bats are considered amplifying hosts of the virus [127,128]. On the other hand, Malsoor virus, which is genetically similar to the severe fever with thrombocytopenia syndrome virus (SFTS, Dabie bandavirus) [129], belongs to the same family and was isolated from the bodies of Indian bats *Rousettus leschenaultii*.

#### 2.5.7. Family *Reoviridae* and *Caliciviridae*

The family *Reoviridae* includes two genera of public health importance: Orbivirus and Rotavirus, which include rotavirus types A, B and C (RVA, RVB, RVC) [123]. Both rotaviruses and noroviruses, which in turn belong to the *Caliciviridae* family, are now some of the most common causes of acute gastroenteritis in humans, and the presence of these viruses has been found in bats around the world [130].

In Europe, rotavirus has been detected in *Miniopterus schreibersii* in Serbia. Based on the genome sequencing results and phylogenetic analysis, the authors proposed classifying this new species as Rotavirus J [131]. Rotaviruses, including RVA, have also been identified in *Myotis* and *Rhinolophus* bats in France, Bulgaria and Germany [132,133] (Table S3). The authors suggest the necessity of their occurrence monitoring, especially in wildlife, due to the risks associated with the proven possibility of the interspecies transmission of rotaviruses [132]. Belonging to the same family as Rotaviruses, the Reoviridae family, Mammalian Orthoreovirus (MRV) has been detected in Germany, Italy, and Slovenia in *Plecotus*, *Myotis*, *Nyctalus*, *Pipistrellus*, *Rhinolophus*, *Vespertilio*, *Tadarida*, *Eptesicus*, and *Miniopterus* bats (Table S3). MRV infections are generally mild or asymptomatic and only in exceptional cases can lead to more severe complications, such as, meningitis and encephalopathy [123,134]. In 2018, another representative of the Reoviridae family, Kadipiro viruscalciv (KDV), was detected in *M. daubentonii* in Denmark for the first time in Europe [135]. The same study also reported on Caliciviruses in *M. daubentonii*. These Caliciviruses scientists had previously detected in fecal samples from *M. daubentonii*, *M. alcathoe*, and *E. serotinus* bats in Hungary and in Denmark (Table S4) [136,137].

#### 2.5.8. Other Viral Pathogens

Bats can also be infected and become reservoirs for many other viruses dangerous to humans, such as Dengue virus (DENV) and influenza A virus (IAV). However, the role of these animals in the spread of viruses is not clear, as the possibility of their transmission to humans has not been proven so far [138]. Previous studies have confirmed that bats harbor highly divergent influenza A-like viruses (IAV), including subtypes H17N10 and H18N11, supporting their role as natural reservoirs of these viruses [139,140,141]. More recent experimental work has further advanced understanding of their molecular biology and replication mechanisms [142], while immunological studies highlight host-specific adaptations that may facilitate long-term virus maintenance in bat populations. Recent findings also highlight potential evolutionary links between bat and avian influenza viruses. In particular, H9 influenza viruses detected in bats appear to be closely related to avian lineages, suggesting past interspecies transmission events. Current evidence supports the hypothesis that the H9 subtype most likely originated in wild birds and was subsequently transmitted to bats, where it established a separate lineage, rather than the reverse scenario. The identification of a novel H19 subtype with mixed avian–bat genomic features further supports the existence of intermediate evolutionary stages and genetic exchange between these host groups [143]. Also, Kandeil et al. [144] detected H9N2-like IAV from Egyptian fruit bats (*Rousettus aegyptiacus*), which is distant from known conventional IAVs.

The lack of replicability in the body of bats, confirmed in the case of DENV, allows bats to be considered epidemiological dead-end hosts [18,42]. Other viruses that have been isolated from bats or confirmed by other methods include Japanese encephalitis virus, Venezuelan equine encephalitis virus, Chikungunya virus, Rio Bravo virus, and Kasokero virus. The bats that carry them are mainly found in Asia, Africa and Central America [42,44,145,146,147,148,149].

### 2.6. The Importance of European Bats Population as a Reservoir of Viruses with Zoonotic Potential—Facts and Questions

The conclusions drawn from the analysis of the data presented above regarding the occurrence and modes of transmission of the most dangerous bat-borne viruses to humans seem to suggest that the problems arising from their presence affect the European continent only to a limited extent. It is worth remembering, however, that apart from climatic and civilizational factors, which certainly reduce the risk of dangerous bat-borne viruses appearing in Europe, the outbreak of a potential pandemic may be determined by factors that are more universal and independent of the specific characteristics of a given region.

Among potentially pathogenic bat-related viruses, the dominant RNA viruses, characterized by high mutation and replication rates that allow them to evolve, expand their variability and under environmental pressure may result in favorable phenotypes [150]. These properties are not only typical to tropical zone viruses and may determine the emergence of new virulent variants also under European climate conditions. The level of population immunity and individual characteristics of the human host, as well as the effectiveness of human-to-human transmission mechanisms, are only partially related to the geographic location of areas harboring animal reservoirs of viral pathogens, while clearly affecting the likelihood of new viral outbreaks. It is therefore reasonable to ask whether the risk of a pandemic caused by bat-associated viruses is significantly higher on any particular continent? And, in this context, how important role can the European bat population play?

#### Bats in Europe

The European continent is currently inhabited by 51 species of bats, belonging to the genera *Rhinolophus* (family *Rhinolophidae*), *Barbastella*, *Eptesicus*, *Hypsugo*, *Myotis*, *Nyctalus*, *Pipistrellus*, *Plecotus*, *Vespertilio* (family *Vespertilionidae*), *Miniopterus* (family *Miniopteridae*), *Tadarida* (family *Molossidae*) and *Rousettus* (family *Pteropodidae*) [151,152]. Except for the fruit-eating *R. aegyptiacus*, all European bats are insectivorous, although *Myotis capaccinii* preys on small fish in addition to insects, and *Nyctalus lasiopterus* preys on birds [153,154]. Each genus and species occupy specific ecological niches, and their distribution depends on available food sources, a place to establish a colony, or thermal conditions typical of the region. There is a clear north–south gradient in the number of bat species, with a higher diversity observed in the southern regions of Europe. Endemic species inhabit islands isolated from the mainland, e.g., Madeira, the Azores, and the Canary Islands (*Pipistrellus maderensis*, *Plecotus teneriffae*, *Nyctalus azoreum*) and Sardinia *Pl. sardus* [155,156,157,158]. Most European bats migrate distances from 10 to 100 km; however, some *Nyctalus* and *Pipistrellus* species are known to migrate more than 1000 km [159]. In view of the observed decline in bat numbers in Europe, conservation initiatives are being established to minimize the level of factors negatively affecting bat populations. The protection of European bats is handled by the EUROBATS organization and the Habitats Directive (EU Council Directive 92/43/EEC) protects all bat species (Agreement on the Conservation of Populations of the Bats) [160,161].

**Figure 4:**
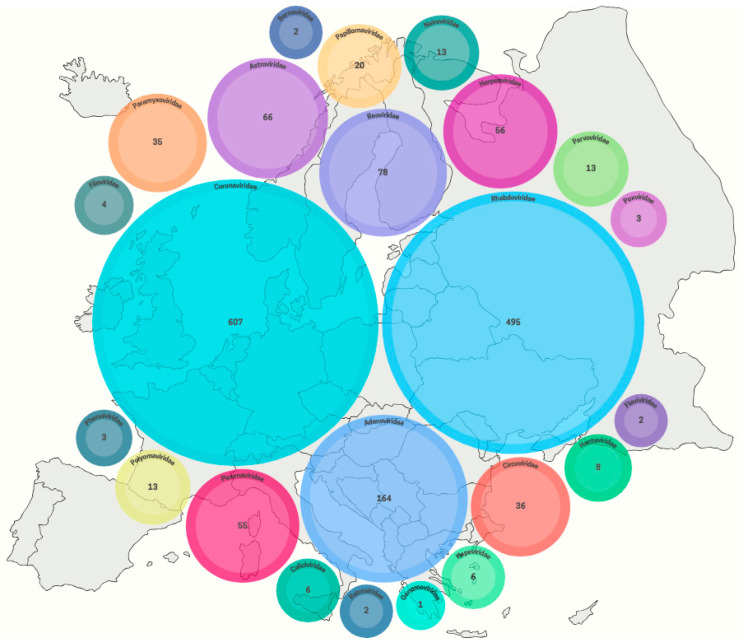
Number of confirmed bat-associated viruses in individual virus families in Europe (data for 1970–2026, from DBatVir database [14]). The virus families are color-coded, and the size of the circle corresponds to the number of isolated viruses. The following viruses were included: dsDNA viruses: *Adenoviridae*, *Herpesviridae*, *Papillomaviridae*, *Polyomaviridae*, *Poxviridae*; dsRNA viruses: *Picobirnaviridae*, *Reoviridae*; *retrotranscribing* viruses: *Hepadnaviridae*, *Retroviridae*; ssDNA viruses: *Genomoviridae*, *Parvoviridae*; ssRNA negative-strand viruses: *Bornaviridae*, *Filoviridae*, *Hantaviridae*, *Paramyxoviridae*; ssRNA positive-strand viruses, no DNA stage: *Astroviridae*, *Caliciviridae*, *Coronaviridae*, *Flaviviridae*, *Hepeviridae*.

Coronaviruses are currently the largest family of viruses detected in European bats (Figure 4). They belong mainly to Alpha- and Betacoronavirus, and have high sequence similarity to SARS-CoV, SARS-CoV-2 and MERS-CoV [34,123]. Considering the criterion of public health risk, a European bat species deserving special attention is *Rhinolophus hipposideros*, in which, among many other Alpha- and Betacoronaviruses, *Sarbecovirus* has also been demonstrated [162] (Table S1). One of the most recent studies of bat-coronaviruses in Europe has proven the presence of SARS-related Betacoronavirus in fecal samples of *R. hipposideros* from areas of Poland. Phylogenetic analysis of the 379 bp-long nucleotide sequence of SARS-related Betacoronavirus isolates identified 96.3–96.5% similarity with a Slovenian coronavirus isolate obtained from horseshoe pit feces in 2008. A comparison with the most genetically similar SARS-CoV-2 Chinese reference coronavirus strain RaTG13 from 2013 showed an identity of 82.0–82.3% [163]. Scientists have detected SARS-related Betacoronaviruses also in Luxembourg in the feces of another representative of this genus, *R. ferrumequinum* [164]. While all SARS-related CoVs have been identified only in bats of the family *Rhinolophidae*, MERS-like coronaviruses have been detected only in representatives of the family *Vespertilionidae*—*Hypsugo savii* and *Nyctalus noctula* in Italy and *Pipistrellus* spp. in Italy, the Netherlands, Russia, Spain, Ukraine, and Romania [165,166]. Figure 4 shows the number of confirmed bat-associated viruses in each viral family in Europe.

Since the COVID-19 outbreak, researchers have described a number of variants of the SARS-CoV-2, pointing out those of particular concern because of their virulence, high transmissibility, lower ability to be neutralized by antibodies, or reduced effectiveness of immunization or therapy. Among five such variants, one, described in late 2021, Alpha (B.1.1.7), originated in Europe (the United Kingdom), while the other four (Beta, Gamma, Delta, Omicron) originated in other continents [167].

SARS-related CoVs detected in European bats do not have alterations in the spike protein that would allow them to infect humans, and results of studies to date have not confirmed cases of direct human infections with this coronavirus from bats [123]. However, as some authors suggest, this may result from inadequate infection control and low detection rates due to the subclinical course of infection or confusion with other diseases [168].

Lyssavirus viruses detected in European bats mainly belong to five species: EBLV-1, EBLV-2, Bokeloh bat lyssavirus (BBLV), West Caucasian bat lyssavirus (WCBV) and Lleida bat lyssavirus (LLEBV) [169] (Table S2). The vast majority among them was EBLV-1, divided into 2 subgroups: EBLV-1a isolated from bats in the Netherlands and Russia, and EBLV-1b found in France, the Netherlands and Spain. EBLV-1 has been identified almost exclusively from serotine bats, primarily *Cnephaeus serotinus*, with the reservoir of EBLV-1b on the Iberian Peninsula being *E. isabellinus* bats [123,170,171,172]. Surveillance data confirm that EBLV-1 remains the predominant bat-acquired lyssavirus circulating in Europe [171]. Recent reports have documented ongoing spillover events beyond bats, including a confirmed infection in a domestic cat in the Netherlands in 2024, representing one of only a few such cases reported in Europe over the past two decades, and highlighting the ability of EBLV-1 to occasionally cross species barriers under natural exposure conditions [173]. EBLV 2, characterized by a much smaller geographic range within the European continent (UK, Netherlands, Finland, Switzerland, Germany), was detected in *Myotis daubentonii* and *M. dasycneme* [37,170]. Other lyssaviruses appear to have more restricted distributions and have been identified only sporadically. These include WCBV and LLEBV, both associated with Schreibers’s bent-winged (*Miniopterus schreibersii*) bat [57,174].

Despite the fact that bats carrying EBLV have adapted to human-transformed environmental conditions and, as synanthropic species, build their habitats in close proximity to humans, the number of reported human cases of rabies caused by these animals in Europe is low [123,175]. This may be attributed to various factors: well-established public health measures—including mandatory reporting systems—widespread rabies vaccination programs in domestic animals, and oral vaccination campaigns targeting wildlife reservoirs. Moreover, active surveillance programs monitoring lyssaviruses in bat populations are implemented across several European countries, including Belgium, France, Germany, Serbia, Slovenia, Spain, Sweden, Switzerland, and the UK [169,170,171,176,177,178,179,180,181,182].

The remaining species of Lyssaviruses have been isolated from European bats and described in the last several years. Researchers have confirmed the presence of *Bokeloh bat lyssavirus* (BBLV) in *Myotis nattereri* in Germany in 2010 [183], France in 2012 [184] and Poland in 2016 [123,185]. In 2002, *West Caucasian bat lyssavirus* (WCBV) was discovered in *Miniopterus schreibersi* in Russia [186]. In 2020, Leopardi et al. [187] reported a WCBV virus, with a high degree of identity to the Russian reference strain, in Italy in the tissues of a rabies-infected cat. Bats were the likely source of infection [187]. In addition to WCBV, bats belonging to the *M. schreibersi* species also carried *Lleida bat lyssavirus* (LLEBV), first isolated in 2011 in Spain [57]. Recent findings, dating back to 2017 from Finland, prove the existence of a new European representative of the genus *Lyssavirus*, detected in the tissues of dead *Myotis brandtii*. Phylogenetic analysis of *Kotalahti bat lyssavirus* indicates that it is closely related to BBLV and EBLV-2 viruses [56].

The presence of paramyxoviruses in Europe does not appear to be a major epidemiological problem to date. Nucleic acids (RNA) of these viruses have been detected in Germany and Italy in bats of the genera *Myotis*, *Nyctalus* and *Pipistrellus*. However, analyses of their genetic sequences has not allowed classification within any of the known genera of *Paramyxoviridae*. Despite the exclusion of their relationship with human-pathogenic paramyxoviruses, the presence in bats inhabiting areas untouched by fruit-eating species suggests the possibility of virus–host coevolution in bats on the European continent. It justifies the need for continuous monitoring of this phenomenon [49,188].

No Marburg or Ebola virus has ever been detected in European bats. The source of the first described MARV human infection and death in 1967 in Germany and former Yugoslavia was contacted from the tissues of infected monkeys. Since then, scientists have registered sporadic cases of MARV in Europe in patients returning from Africa, where they were exposed to direct contact with bats, or in employees of specialized laboratories [123,189]. In the case of EBOV, isolated cases of Ebola in Europe have emerged in Spain and the Netherlands from people who previously worked with infected patients from outside Europe or who resided in affected areas [190].

Another Filovirus carried by bats is endemic to Europe Lloviu virus (LLOV), belonging to the genus *Cuevavirus*. This virus caused the mass death of Schreiber’s bats, shrinking their colony size (*Miniopterus schreibersii*) in France, Spain, and Portugal in 2002. The results of the studies conducted at this time, combined with conclusions drawn after the appearance of subsequent LLOV outbreaks in bats in Hungary (2013, 2016, 2017), authorized the conclusion that LLOV infection in bats is not subclinical. The fact that the Lloviu virus was only found in dead bats supports this thesis [123,191]. Nonetheless, the latest 2018–2020 studies conducted in Hungary, which included RT-PCR analyses of samples also taken from live, disease-free bats, confirmed the virus presence in the blood of these animals. They also suggest that Schreiber’s bats are not only hosts, but also a natural reservoir of the virus in Europe. The migratory capabilities of Schreiber’s bats, as well as the virus’ demonstrated ability to infect monkey and human cells in vitro tests, provide important arguments for the thorough investigation and monitoring of LLOV both within Europe and adjacent areas [120].

European hantaviruses cause hemorrhagic fever with renal syndrome (HFRS) in humans. Scientists have detected a new hantavirus Brno virus (BRNV) in the common noctule (*Nyctalus noctula*), in the Czech Republic [126]. A study of Dafalla et al. [125] confirmed its presence in bats of the same species from Poland, Austria and Germany.

Viruses belonging to the *Phenuiviridae* family most frequently detected in bats from Germany include those identified in *Pipistrellus nathusii* Bavarian bat lalavirus (BblV) and Munich bat lalavirus (MblV) and those detected in *Cnephaeus nilssonii*, Zwiesel bat banyangvirus, ZbbV (genus *Bandavirus*), which are closely related to Malsoor virus [48,192]. On the other hand, the results of a 2006–2018 study in Spain on the presence of the Toscana virus, TOSV (genus *Phlebovirus*), in bats common in the Mediterranean region, indicate a negligible role for these animals in the natural cycle of this virus, transmitted by sand flies [193]. The results of an Italian study confirming the isolation of TOSV from the brain of *Pipistrellus kuhlii* are the only ones that indicate a possible link between bats and the virus. However, according to some scientists, the precision of the performed analyses may raise some doubts (possibility of cross-contamination of samples) [123,194].

In Europe, in addition to the viruses described above, the Usutu virus (family *Flaviviridae*) was detected in 2013 in the brain of *Pipistrellus pipistrellus* bats in Germany. In humans it generally causes asymptomatic or mild infections, but can occasionally lead to neurological disorders, including encephalitis and meningitis. In the case of this mosquito-borne virus, however, bats act more as incidental or dead-end hosts [195,196]. Scientists have also focused on viruses of the *Nairoviridae* family, detected in bats in Germany—Berlin bat Nairovirus and Wittenau bat Nairovirus in *P. pipistrellus*, and Issyk-Kul virus (ISKV) in *Cnephaeus nilssonii*. Issuk-Kul virus was also discovered in 2018 in Sweden in *Carios vespertilionis* ticks that fed on the blood of the bat *Pipistrellus pygmaeus*. ISKV, identified mainly in Asia, is transmitted by ticks and can cause fever, headache, muscle aches and nausea in humans [197]. A bat norovirus, Ahun Nairovirus was also detected in lungs of *Pipistrellus pipistrellus* and *Myotis mystacinus* in France [198].

For a virus circulating in wildlife to cause a human pandemic, it must overcome several biological barriers. It needs the ability to infect human cells efficiently, replicate in the human host, and spread effectively between people, often through the respiratory route [199,200]. The COVID-19 pandemic illustrates this process well: SARS-CoV-2 evolved a spike protein capable of binding efficiently to the human ACE2 receptor, which enabled rapid human-to-human transmission [201,202].

Although European bats host a variety of RNA viruses, including coronaviruses and lyssaviruses, studies indicate that most of these viruses are poorly adapted to infect human cells [17,203]. Many bat coronaviruses detected in Europe show limited compatibility with the human ACE2 receptor, which reduces their zoonotic potential [204,205].

One of the few known documented zoonotic infections in humans is a rabies-like disease caused by European bat lyssavirus (EBLV). However, human cases occur only in exceptional circumstances, usually after direct contact with infected bats such as bites or scratches [206].

### 2.7. The Frequency of Human–Bat Interactions

The potential for bats to transmit viral diseases is an undisputed fact and represents an epidemiological risk that should not be underestimated. At the same time, it should be emphasized that the mere presence of a bat in the human environment—even one that serves as a reservoir for a virus—does not necessarily result in infection or viral disease outbreak.

Estimating the epidemic potential of viruses is undoubtedly challenging due to the level of complexity of the problem. Therefore, it is necessary to consider all the factors that increase the likelihood of optimal conditions for mass infections. These factors include the impact of a number of external factors that increase the virus virulence, but also possible modifications occurring in its structure, which can change the nature of the human–virus relationship from neutral to parasitic. Understanding how viruses are transmitted in the environment and the role of reservoir organisms and intermediate hosts is also crucial in determining the real level of epidemic risk.

When analyzing the risk of virus transmission from an animal host to humans, it should be emphasized that the unique predisposition of bats as virus carriers is of epidemiological significance only if many other conditions are met. Infection caused by bat-associated viruses can only occur when there is human contact with a bat that is a reservoir of pathogenic viruses or with its secretions.

For most inhabitants of Europe, direct contact with bats is rather incidental. The sporadic nature of bat–human interactions largely results from the considerable distance that usually separates bat habitats from human settlements. However, bat colonies inhabiting residential buildings and other human-used structures may increase the frequency of direct contact. Bat populations living far from human habitats do not pose a threat to public health; on the contrary, human interference with the population structures of these animals may significantly increase this threat [207].

However, many forms of human activity often lead to a reduction in the distance between bats and humans. Transformations primarily involving the deforestation of increasingly large areas for agricultural purposes result in the destruction of natural bat habitats and promote the relocation of bats to the vicinity of human settlements [208]. As a consequence, the likelihood of interspecies contact between virus reservoirs and humans increases, potentially facilitating viral mutations that enable adaptation to new hosts [209,210].

In contrast to other continents, deforestation in Europe is limited to a few regions and does not constitute a key factor increasing the frequency of human–bat contact. However, forests provide the principal habitat for European bats, which underscores the importance of maintaining sustainable forest management practices [156]. European environmental policies aimed at preserving adequate forest cover or systematically restoring it may limit bat migration to human settlements and reduce the likelihood of contact.

Urbanization, which is progressing in European countries, is also an element of environmental pressure, leading to the destruction of natural habitats for bats and forcing them to move to areas increasingly close to humans. In addition to shortening the human–bat distance, urbanization can also lead to the depletion of the biodiversity of bat populations in an area, for example by filtering out light-sensitive species. However, light pollution may provoke migration of bats to other locations without significantly affecting their population. Bats can adapt to new sites by creating near-optimal habitat conditions for themselves. On the other hand, it has been suggested that the greater number of insects that congregate around intensely lit areas at night may make it easier for bats to forage and positively affect their functioning in urbanized regions, resulting in an increase in their population and a higher probability of contact with a human or other organism that may act as an intermediate host [211]. This, combined with the population density typical of large metropolitan areas, can increase the risk of new outbreaks of bat-associated viruses.

Among the human-dependent factors that can promote such viral zoonotic infections are also socio-cultural customs specific to different countries in different parts of the world. In many countries of Asia, Africa, or South America, the consumption of bat meat is widespread, hunting of these mammals is arranged, and wild animals, including bats, are sold and stored in direct contact at markets (wet markets). Unfortunately, enacting restrictive laws to curb these practices can be counterproductive, causing the trade in wild animals and their meat to take place through illegal channels with no way to control them [212]. In Europe, the risk of such zoonotic spillover events is generally considered lower due to stricter sanitary regulations, controlled wildlife trade, and the absence of widespread bat consumption.

For many bat-associated zoonotic viruses, spillover to humans involves intermediate hosts that act as amplification reservoirs. However, such transmission pathways are uncommon in Europe due to the absence or limited presence of relevant intermediate hosts (e.g., dromedary camels in the case of MERS-CoV). Although pigs, which serve as amplification hosts for NiV and HeV, are common farm animals in Europe, farming practices and biosecurity measures generally limit their contact with wild bats. Moreover, the frequency of human contact with potential host animals is lower in Europe compared with Asia or Africa [130,213]. A comprehensive review of the available literature and surveillance data was conducted to assess the occurrence of bat-associated viral infections in humans in Europe. A total of four confirmed human cases of such infections were identified, all attributable to rabies caused by EBLV. No documented cases of human infection with other bat-borne viruses (including coronaviruses, paramyxoviruses, hantaviruses, filoviruses, or others) have been reported in Europe to date, and no reliable evidence is available. In 1977 (Ukraine), a patient developed fatal encephalitis following a bat bite (infection with EBLV-1 was confirmed by virus isolation from the central nervous system). In 1985 (Russia, Voroshilovgrad/Belgorod region), a fatal case of rabies occurred after a bat bite, with laboratory confirmation of EBLV-1 infection. In the same year, in Finland, a 30-year-old bat researcher with a history of repeated bat bites died of rabies and was diagnosed post-mortem with EBLV-2 infection. This was the first laboratory-confirmed case of the EBLV-2 virus in humans. Finally, in 2002 (United Kingdom, Angus, Scotland), a 56-year-old bat conservationist with repeated minor exposures (bites and scratches) developed rabies and died (EBLV-2 infection was confirmed by serological testing and virus isolation) [94,214].

### 2.8. Climate and Viral Dynamics in Bat Populations

Climate plays an important role in shaping the ecology of bats, the diversity of bat-associated viruses, and the potential for interactions between bats and humans. The temperate climate characteristic of most of Europe is associated with relatively lower bat species richness compared with tropical regions. These ecological conditions are generally linked to lower viral diversity and may reduce the probability of efficient transmission of bat-associated viruses to humans [25,215,216,217].

Temperature also affects the stability and persistence of viruses in the environment outside their hosts. In cooler temperate climates, some viruses may remain viable for longer periods under environmental conditions. McKee et al. [101] showed that colder winter temperatures were associated with more spillover events of NiV compared with relatively warmer periods. However, this potential advantage for viral persistence is often offset by lower host activity and reduced contact rates among animals. Colder winters in Europe have historically restricted the distribution of many bat species, resulting in a relatively limited number of virus–host combinations compared with tropical regions. Low temperatures may also reduce the activity of potential viral vectors [218]. In contrast, tropical ecosystems support year-round activity of bat populations, which facilitates sustained viral circulation and transmission [219].

Seasonality in temperate regions further influences viral dynamics through winter hibernation. In these environments, hibernation represents a key ecological adaptation that allows bats to survive seasonal food scarcity and unfavorable climatic conditions [220]. Many European bat species enter prolonged hibernation during the colder months, during which body temperature and metabolic rate decrease substantially and interactions between individuals become less frequent. These physiological and behavioral changes may significantly influence viral replication, persistence, and transmission within bat populations.

Climate plays a key role in determining the geographic distribution of bat species and the diversity of host–virus associations. However, ongoing climate warming may alter these patterns. Extreme climatic events, which are often the result of irresponsible human activity, can significantly affect bat populations. Prolonged drought periods and violent atmospheric phenomena, as well as accidental or deliberate fires and the destruction of bat-inhabited caves, not only pose a risk to bats’ survival but also lead to the loss of foraging and roosting sites [221,222]. Reducing the availability of existing bat habitat forces, in turn, their migration to new areas. Accompanying changes in abundance and species composition may in turn lead to the modification of host–pathogen interactions toward the evolution of viruses that increase and disease causing activity, or by refining those already in place and creating new pathways for their transmission [223].

Range shifts observed in response to climate change may result in an increase in the abundance and biodiversity of bats and, consequently, the pathogens they carry. In recent years, the increase in bat species richness has occurred primarily in Central Africa, Central and South America, but also in areas of Southern China, Myanmar and Laos, locations associated with the bat-associated SARS-CoV-1 and SARS-CoV-2 [221]. However, given the universality and intensification of the scale of climatic phenomena, it can be assumed that similar relationships, albeit on a rather smaller scale, may also apply to some areas of Europe.

### 2.9. Globalization and Mobility

The emergence and spread of infectious diseases are strongly influenced by globalization and modern human mobility. Even if a zoonotic virus initially appears in a limited geographic area, it can spread rapidly when introduced into regions with intensive international connections. Europe is highly connected through international transport networks, meaning that a newly emerging virus elsewhere could be rapidly introduced, as demonstrated during the COVID-19 pandemic [224,225].

Generally, the likelihood of a locally emerging bat-origin epidemic in Europe appears lower than in Asia, Africa, or parts of South America, where high biodiversity, wildlife trade, and frequent human–wildlife interactions increase the probability of zoonotic spillover. Nevertheless, once a pathogen appears anywhere in the world, Europe’s strong transport connectivity may facilitate its rapid arrival and spread [226,227].

### 2.10. Scientific Capacity and Public Health Preparedness

The risk that viruses transmitted by bats could trigger a widespread epidemic also depends on the level of scientific capacity, surveillance systems and healthcare infrastructure in a given region. In Europe, advanced biomedical research networks and well-developed epidemiological databases support early detection and monitoring of emerging zoonotic pathogens, including viruses associated with bats [10,228]. Nevertheless, the available data from Europe remain limited in comparison with studies conducted in recognized hotspots of emerging infectious diseases. This discrepancy partly reflects a persistent assumption that Europe represents a lower-risk region due to its comparatively lower bat biodiversity and the absence of major documented spillover events. As a consequence, virological surveillance of European bat populations has historically been less intensive than in other parts of the world. Such a research bias may lead to an underestimation of both the diversity and prevalence of bat-associated viruses in Europe and may reduce the likelihood of early detection of newly emerging viral agents [54,58].

Europe also benefits from coordinated public health institutions and strong healthcare systems that enable rapid diagnosis, outbreak investigation, and implementation of control measures such as isolation, vaccination, and contact tracing [36,229]. Additionally, surveillance programs for bat-borne viruses operate across many European countries using a One Health approach, sometimes described as “One Bat” initiatives, which integrate bat ecology, veterinary diagnostics, and public health monitoring to track pathogens such as European bat lyssaviruses. These systems include both passive surveillance of sick or dead bats and targeted monitoring of wild populations [230].

A potential weak point of the relatively effective healthcare system in Europe may be the limited preparedness of local medical services for a potential pandemic, resulting from the absence or low frequency of emerging pathogens in the region. This may lead to challenges in the early diagnosis of unusual clinical symptoms and in linking them to their specific etiological agents. Furthermore, in the event of a large-scale outbreak, additional difficulties may arise in ensuring the rapid availability of sufficient quantities of vaccines or therapeutic agents, which are essential for an effective response to a mass public health threat [231].

### 2.11. Measures and Strategies to Minimize the Risk of Virus Transmission by Bats

Reducing the risk of pathogenic virus transmission by bats requires large-scale, multidirectional efforts. These should involve the development of specific strategies focusing, among other things, on maintaining the natural distance between bats and humans. An important component of bat conservation strategies is the protection of their habitat networks, which are essential for foraging, mating, reproduction and hibernation, as well as protecting these animals from being killed [221,232]. According to the International Union for Conservation of Nature, 17.5% of bat species are considered critically endangered, endangered or vulnerable, due to the high sensitivity of these mammals to stress factors, especially those of an anthropogenic nature [233,234]. Therefore, it is necessary to develop and implement guidelines for responsible forest management. They should focus on halting this process and raising the level of investment in afforestation and reforestation across the globe [208]. In areas affected by unsustainable forest management practices and regions where this phenomenon has not yet reached alarming levels, it is essential to implement actions that promote sustainable agricultural practices to minimize disruptions to bat habitats.

Bats play an incredibly relevant role in ecosystems, such as crop pest control, pollination, and nutrient redistribution. Therefore, it is crucial to implement programs that raise public awareness about the ecological significance of bats. Implementation of these programs should be focused on promoting sound knowledge conveyed in a way that does not evoke unwarranted emotions. This will avoid perpetuating negative stereotypes about bats in local communities and rationalize the relationship between these animals and humans. Knowledge of the role humans play in the potential spread of bat-associated viruses may contribute to informed decisions about avoiding contact with bats, but will not provoke aggressive behavior [235].

Legal regulations, implemented with the intention of safeguarding endangered species, unfortunately restricts the ability to acquire the materials required for conducting rigorous scientific research. As a result, one priority in areas such as monitoring the frequency of occurrence and comprehensive characteristics of bat-borne viruses is the necessity of optimizing existing and developing new minimally invasive sampling methods.

Non-invasive methods allow screening and assessment of the viral shedding, and potential risk of spread. Regular monitoring of sampled material for the infectious agent would also provide an essential source of information on any unusual cases that could lead to human disease or even epidemics. Viral shedding can be affected by both internal and external factors, such as stress, starvation, weaning, loss of maternal antibodies, and pregnancy [236]. The variable prevalence of viruses in material samples, observed especially for pathogenic viruses that cause acute infection and a prolonged immune response, makes detection of these microorganisms difficult. In contrast, studies have shown that euthanasia, an invasive method, does not increase the likelihood of virus detection but can negatively affect bat populations, especially those with low sizes [237]. Taking a sample of material (serum, saliva, feces) from animals, even without killing, requires capturing and using sedatives, which can induce behavioral changes and affect its health and survival, as has been shown in some studies conducted on birds [238]. Invasive collection also increases the risk of infection for the person performing the test. An alternative method of testing bat blood of *Bartonella* spp., *Polychromophilus* spp. and *Trypanosoma* spp., with reduced invasiveness, was used by Szentiványi et al. [239]. These researchers used hematophagous ectoparasitic flies that often feed on the blood of their hosts and determined the presence of microorganisms in collected samples using molecular methods. However, further research on the feasibility of using ectoparasites as a substitute for the invasive blood collection method is needed.

Acoustic recording of bats, involving recording the sounds of echolocation signals, allows monitoring of their biodiversity. This method, together with laser technology (Light Detection and Ranging; LIDAR) can be used to assess the occurrence, abundance and mortality of bats and determine their activity patterns [240]. Such information may be relevant to epidemiology and the study of transmission of viruses responsible for disease in humans. For example, Amman et al. [241] found that the risk of Marburg virus infection in humans increases twice a year and coincides with the peak breeding season of fruit bats *Rousettus aegyptiacus*—the likely natural reservoir host of the virus. Thus, acoustic monitoring can indirectly enhance surveillance and control of infectious diseases [242].

Non-invasive methods include testing bat guano, their urine, or swabs taken from the mouths of dead animals. The choice of sampled material affects the detected virion due to the varying tissue tropism of viruses and the different transmission routes. Hence, testing should target a specific type of specimen, increasing the efficiency of pathogen detection [243].

Feces are the most commonly tested material for monitoring pathogens inhabiting bat organisms, primarily due to the ease of collecting this sample type. A longitudinal sampling strategy, examining the same individual at different time points, is rarely used. In bat-borne disease research, the so-called “sampling under the roost,” using plastic sheets, is primarily used [244]. In their work, Giles et al. [245] presented an optimal collection sampling strategy using increased number of small sheets distributed over the roost area. This method has high sensitivity and specificity and lower cost of testing resulting from minimizing the number of tests performed through a pooling strategy. Urine samples can also be collected on plastic sheets in bat foraging or hibernation areas. Urine can be sampled from leaves, after spontaneous urination, or snow, in frozen form. Factors limiting the use of urine for research are sunlight, contamination of urine with feces, or dilution with snow.

Given that for more than half of virus families there is no data on the optimal type of material collected for their detection, metagenomic analyses, including high-throughput methodologies (HTS), are worth consideration. Such pathogen-specific untargeted assays can detect the genetic material of any virus present in a sample without the need for primer design, and reveal new, previously unexplored viral lineages or families [246,247]. Currently, metagenomic Next-Generation Sequencing (NGS) constitutes approximately 30% of all virus sequence data isolated from bats [17]. NGS are ultrasensitive [246]. However, the presence of viral genetic material in samples such as feces may not necessarily indicate the possibility of viruses of crossing the intestinal wall and infecting. Simultaneous sampling from the environment and other animals and their metagenomic analysis may be helpful [248].

A disadvantage of non-invasive sampling methods is the risk of overestimating the prevalence of virus-source bats, since the pooled sample consists of samples from an unknown and variable number of individuals. Highly sensitive molecular biology methods for pooled samples allows very good surveillance of the risk of disease emergence in the case of low pathogen prevalence. However, it does not provide the capability to differentiate samples from individual specimens [245]. Therefore, further efforts should be taken to develop improved sampling methods that minimize bats exposure to stress.

## 3. Conclusions

Bats, as major reservoirs of diverse RNA viruses, are therefore frequently considered potential sources of such an emerging threat. The concept of “Disease X”, introduced by the World Health Organization (WHO), refers to the possibility that a future pandemic may be caused by a currently unknown pathogen with zoonotic origin [249].

Available evidence suggests that the probability of a large-scale epidemic caused by bat-borne viruses emerging directly in Europe is lower than in other regions of the world such as Asia or Africa. This is directly related to climatic conditions, lower bat species diversity, limited human–bat interactions, and well-developed healthcare systems and surveillance networks. Furthermore, as global analyses indicate, the epidemiological risk in Europe is more closely linked to rodent-borne zoonoses than to bat-borne ones, while predicted hotspots of bat-borne diseases are concentrated mainly in Africa.

Nevertheless, a lower probability does not eliminate the risk. Bat-associated RNA viruses evolve rapidly through mutation and recombination, which may lead to the emergence of new variants capable of infecting humans, regardless of the continent they live on. Genomic analyses suggest that regions such as Southeast Asia harbor numerous still-undiscovered bat species, some of which may host previously unknown pathogenic viruses [250]. Both undiscovered and newly emerging viral strains can easily spread across the globe. While Europe appears to have a lower risk of primary spillover from local bats compared with other continents, its dense transport networks make it highly vulnerable to imported infectious diseases.

Therefore, continuous monitoring of bat populations and their viruses, combined with expanded surveillance systems and stronger international scientific collaboration, is essential. Rapid data sharing and coordinated global research efforts will be crucial for the early detection and prevention of future zoonotic outbreaks, including the potential emergence of “Disease X”.

## Data Availability

No original data were presented in this article.

## Supplementary Materials

The following supporting information can be downloaded at: https://www.mdpi.com/article/10.3390/v18050535/s1, Table S1: Cases of coronavirus isolation from bats in Europe; Table S2: Cases of Rhabdovirus isolation from bats in Europe; Table S3: Cases of Reoviridae family viruses isolation from bats in Europe; Table S4: Cases of Calciviridae family viruses isolation from bats in Europe.
