# Supplementary material for: Bat-Borne Viruses and Pandemic Risk: Could Europe Be an Emergence Hotspot?
Source: Viruses. 2026 May 2;18(5):535. doi: 10.3390/v18050535 (PMC13211416; doi:10.3390/v18050535)
Supplement: Supplementary file 1 [file viruses-18-00535-s001.zip › Skowron et al. - Table S1.pdf]

Supplementary Table S1. Cases of coronavirus isolation from bats in Europe.

Bat species	Sample type	Collection year	Sampling country	Viruses
Eptesicusisabellinus	Feces	2007	Spain	BetacoronavirusE.isa/M/Spain/2007
Eptesicusnilssoni		2014	Finland	Bat betacoronavirusBtCoV/601_14/E.nil/FIN/2014
Eptesicusserotinus		2014	Denmark	Bat coronavirus BtCoV/7541-24/E.ser/DK/2014
	Swabs	2012	Italy	BetacoronavirusEptesicus/13RS384_26/Italy/2012
Hypsugosavii	Tissue, mix=viscera (lung, heart, spleen and liver) and intestine homogenized	2011, 2020, 2021	Italy	Middle East respiratory syndrome coronavirus Bat-CoV/H.savii/Italy/206645-40/2011<br>BetacoronavirusH.sav/Italy/206645-40/2011<br>Orthocoronavirinae sp. 247433-2<br>Orthocoronavirinae sp. 378052-11<br>Orthocoronavirinae sp. 378052-3<br>Orthocoronavirinae sp. 228741-35-int<br>Orthocoronavirinae sp. 228741-35-vis<br>Orthocoronavirinae sp. 297348-34-int<br>Orthocoronavirinae sp. 297348-34-vis
	Feces	2007	Spain	Alphacoronavirus H.sav/L/Spain/2007<br>BetacoronavirusH.sav/J/Spain/2007
Miniopterusschreibersii	Feces	2008	Bulgaria	Bat coronaviruses:<br>BtCoV/BR98-14/BGR/2008<br>BtCoV/BR98-18/BGR/2008<br>BtCoV/BR98-30/BGR/2008<br>BtCoV/BR98-31/BGR/2008<br>BtCoV/BR98-37/BGR/2008<br>BtCoV/BR98-40/BGR/2008<br>BtCoV/BR98-52/BGR/2008<br>8 BtCoV/BR98-53/BGR/2008<br>BtCoV/BR98-55/BGR/2008
	Feces	2016	Croatia	Bat coronavirus 14/G-MsMm
	Feces, mix=intestinal samples from carcasses of bats	2013, 2014	France	Alphacoronavirus sp. CS131010<br>Alphacoronavirus sp. FRA_EPI8_Misch34_7H_P11<br>Alphacoronavirus sp. FRA_EPI8_Misch35_8D_P11<br>Alphacoronavirus sp. FRA_EPI8_Misch37_9A_P11
		2014	Georgia	Colacovirus sp. GE_CoV68_Min_sch<br>Colacovirus sp. GE_CoV78_Min_sch<br>Myotacovirus sp. GE_CoV61_Min_sch

				Myotacovirus sp. GE_CoV64_Min_sch<br>Myotacovirus sp. GE_CoV67_Min_sch
	Swabs	2021	Italy	Bat coronavirus 58
	Feces, swabs	2022	Portugal	Bat coronavirus AN25<br>Bat coronavirus F25<br>Bat coronavirus F8
	Feces, swabs	2004, 2011, 2020	Spain	Alphacoronavirus M.sch/A/Spain/2004<br>Alphacoronavirus sp. FRA_EPI7_Misch5.1132_4A_P1<br>Bat alphacoronavirus 78<br>Bat alphacoronavirus 82_1<br>Bat alphacoronavirus BT40<br>Bat alphacoronavirus BT42<br>Bat alphacoronavirus BT82<br>Bat alphacoronavirus BT83_2<br>Bat alphacoronavirus BT83_3<br>Bat alphacoronavirus BT83_4<br>Bat alphacoronavirus BT83_5<br>Bat alphacoronavirus BT87<br>Bat betacoronavirus BT68
Myotisbechsteinii	Feces	2007, 2013	Germany	Bat coronavirus M.bec/Germany/D6.6/2007<br>Bat coronavirus M.bech/GER/8355/2014 2001
Myotisblythii		2014	Georgia	Colacovirus sp. GE_CoV119_Myo_bly<br>Colacovirus sp. GE_CoV120_Myo_bly<br>Colacovirus sp. GE_CoV122_Myo_bly<br>Colacovirus sp. GE_CoV124_Myo_bly<br>Colacovirus sp. GE_CoV125_Myo_bly<br>Colacovirus sp. GE_CoV127_Myo_bly<br>Colacovirus sp. GE_CoV129_Myo_bly<br>Merbecovirus sp. GE_CoV142_Myo_bly<br>Merbecovirus sp. GE_CoV143_Myo_bly<br>Merbecovirus sp. GE_CoV145_Myo_bly<br>Myotacovirus sp. GE_CoV121_Myo_bly<br>Sarbecovirus sp. GE_CoV101_Myo_bly<br>Sarbecovirus sp. GE_CoV102_Myo_bly<br>Sarbecovirus sp. GE_CoV105_Myo_bly<br>Sarbecovirus sp. GE_CoV106_Myo_bly<br>Sarbecovirus sp. GE_CoV107_Myo_bly<br>Sarbecovirus sp. GE_CoV146_Myo_bly<br>Sarbecovirus sp. GE_CoV151_Myo_bly
	Swabs	2012	Italy	AlphacoronavirusMyotis/13rs384_31/It-aly/2012
		2004	Spain	Alphacoronavirus M.bly/B/Spain/2004
Myotisbrandtii	Feces	2015, 2016	Finland	Bat alphacoronavirus BtCoV/001_15/M.bra/FIN/2015<br>Bat alphacoronavirus BtCoV/008_16/M.bra/FIN/2016

		2015	Russia	Bat coronavirus Bat-CoV/M.brandtii/Russia/MOW15-27/1
Myotiscapaccinii	Feces, swabs	2015, 2020	Spain	Alphacoronavirus sp. SPA_EPI5_Myocap11_2C_p25<br>Alphacoronavirus sp. SPA_EPI5_Myocap19_3C_p25<br>Alphacoronavirus sp. SPA_EPI5_Myocap31_4G_p25<br>Alphacoronavirus sp. SPA_EPI5_Myocap38_5F_p25<br>Alphacoronavirus sp. SPA_EPI5_Myocap40_5H_p25<br>Alphacoronavirus sp. SPA_EPI5_Myocap90_12B_p25<br>Bat alphacoronavirus BT122_1<br>Bat alphacoronavirus BT122_2<br>Bat alphacoronavirus BT122_3<br>Bat alphacoronavirus BT122_4<br>Bat alphacoronavirus BT122_5<br>Bat alphacoronavirus BT123
Myotis crypticus	Mix, tissue	2021	Italy	Orthocoronavirinae sp. 297348-29-int<br>Orthocoronavirinae sp. 297348-29-vis
Myotisdasycneme	Feces	2015, 2016	Denmark	Bat coronavirus BtCoV/21164-5/M.das/DK/2015<br>Bat coronavirus BtCoV/18799-20/M.das/DK/2016<br>Bat coronavirus BtCoV/18802-1/M.das/DK/2016
		2007	Germany	Bat coronavirus M.das/Germany/D2.2/2007<br>Bat coronavirus M.das/Germany/D3.10/2007<br>Bat coronavirus M.das/Germany/D3.15/2007<br>Bat coronavirus M.das/Germany/D3.28/2007<br>Bat coronavirus M.das/Germany/D3.3/2007<br>Bat coronavirus M.das/Germany/D3.33/2007<br>Bat coronavirus M.das/Germany/D3.38/2007<br>Bat coronavirus M.das/Germany/D3.4/2007<br>Bat coronavirus M.das/Germany/D3.5/2007<br>Bat coronavirus M.das/Germany/D3.6/2007<br>Bat coronavirus M.das/Germany/D5.17/2007
		2006, 2007, 2008	Netherlands	CoronavirusM.das/VM105/2006/NLD<br>CoronavirusM.das/VM34/2006/NLD<br>CoronavirusM.das/VM2/2007/NLD<br>CoronavirusM.das/VM3/2007/NLD<br>CoronavirusM.das/VM62/2007/NLD<br>CoronavirusM.das/VM7/2007/NLD<br>CoronavirusM.das/VM73/2007/NLD

				CoronavirusM.das/VM84/2007/NLD Corona-virusM.das/VM284/2008/NLD
Myotis daubentonii	Feces	2013, 2014, 2015, 2016, 2018	Denmark	Bat coronaviruses:BtCoV/OV13-11/M.dau/DK/2013<br>BtCoV/OV13-16/M.dau/DK/2013<br>BtCoV/OV13-18/M.dau/DK/2013<br>BtCoV/OV13-19/M.dau/DK/2013<br>BtCoV/OV13-24/M.dau/DK/2013<br>BtCoV/OV13-25/M.dau/DK/2013<br>BtCoV/OV13-31/M.dau/DK/2013<br>BtCoV/OV13-42/M.dau/DK/2013<br>BtCoV/13585-12/M.dau/DK/2014<br>BtCoV/13585-35/M.dau/DK/2014<br>BtCoV/13585-36/M.dau/DK/2014<br>BtCoV/13585-41/M.dau/DK/2014<br>BtCoV/13585-42/M.dau/DK/2014<br>BtCoV/13585-52/M.dau/DK/2014<br>BtCoV/13585-58/M.dau/DK/2014<br>BtCoV/13585-60/M.dau/DK/2014<br>BtCoV/21162-1/M.dau/DK/2015<br>BtCoV/21162-2/M.dau/DK/2015<br>BtCoV/21162-4/M.dau/DK/2015<br>BtCoV/21162-6/M.dau/DK/2015<br>BtCoV/21162-7/M.dau/DK/2015<br>BtCoV/21162-8/M.dau/DK/2015<br>BtCoV/21164-2/M.dau/DK/2015<br>BtCoV/21164-3/M.dau/DK/2015<br>BtCoV/21164-6-alt/M.dau/DK/2015<br>BtCoV/21164-6/M.dau/DK/2015<br>BtCoV/18799-11/M.dau/DK/2016<br>BtCoV/18799-2/M.dau/DK/2016<br>BtCoV/18799-25/M.dau/DK/2016<br>BtCoV/18799-33/M.dau/DK/2016<br>BtCoV/18799-34/M.dau/DK/2016<br>BtCoV/18799-38/M.dau/DK/2016<br>BtCoV/18799-52/M.dau/DK/2016<br>BtCoV/18799-52/M.dau/DK/2016, PanCoV A<br>BtCoV/18799-54/M.dau/DK/2016<br>BtCoV/18799-56/M.dau/DK/2016<br>BtCoV/18799-56/M.dau/DK/2016, PanCoV A<br>BtCoV/18799-60/M.dau/DK/2016<br>BtCoV/18799-60/M.dau/DK/2016, PanCoV A<br>BtCoV/18799-62/M.dau/DK/2016<br>BtCoV/OV-157/M.dau/DK/2018
		2014, 2016	Finland	Bat alphacoronaviruses:<br>BtCoV/416_14/M.dau/FIN/2014<br>BtCoV/606_14/M.dau/FIN/2014

				BtCoV/012_16/M.dau/FIN/2016<br>BtCoV/020_16/M.dau/FIN/2016
	Feces	2014, 2015, 2017, 2018, 2019	France	Alphacoronavirus sp. FRA_EPI4_Mydau4_E7_P6<br>Alphacoronavirus sp. FRA_EPI4_Myodau2.4645_4G_P5<br>Alphacoronavirus sp. FRA_EPI4_3915_8E_P30<br>Bat alphacoronavirus Swarming2017_CL31F<br>Bat alphacoronavirus Swarming2017_CL56F<br>Bat alphacoronavirus Swarming2017_FT213F<br>Bat alphacoronavirus Swarming2017_FT61F<br>Bat alphacoronavirus Swarming2018_CL34F<br>Bat alphacoronavirus Swarming2018_FT18.163F<br>Bat alphacoronavirus Pag_T10B3_Jun<br>Bat alphacoronavirus Pag_T12B2_Oct<br>Bat alphacoronavirus Pag_T15B1_Jun<br>Bat alphacoronavirus Pag_T15B2_Oct<br>Bat alphacoronavirus Pag_T17B2_Oct<br>Bat alphacoronavirus Pag_T18B2_Jun<br>Bat alphacoronavirus Pag_T19B2_Oct<br>Bat alphacoronavirus Pag_T22B2_Jun<br>Bat alphacoronavirus Pag_T2B1_Jun<br>Bat alphacoronavirus Pag_T6B2_Oct<br>Bat alphacoronavirus Pag_T6B3_Aug
		2007, 2008	Germany	Bat coronavirus M.dau/Germany/D7.3/2007<br>Bat coronavirus M.dau/Germany/D8.32/2007<br>Bat coronavirus M.dau/Germany/D8.38/2007<br>Bat coronavirus M.dau/Germany/D8.42/2007<br>Bat coronavirus M.dau/Germany/D8.45/2007<br>Bat coronavirus M.dau/Germany/D8.46/2007<br>Bat coronavirus NM98-62/GER/2008<br>BtCoV/NM98-62/GER/2008
		2013	Hungary	Coronavirus BtCoV/V16/HUN/2013
		2007, 2008	Netherlands	CoronavirusM.dau/VM222/2007/NLD<br>CoronavirusM.dau/VM303/2008/NLD<br>Coronavirus M.dau/VM361/2008/NLD
		2020, 2021	Poland	Bat alphacoronavirus BtCoV_148<br>Bat alphacoronavirus BtCoV_69
		2007	Spain	Alphacoronavirus M.dau/H/Spain/2007
		2020	Sweden	Bat alphacoronavirus BtCoVBat-Guano15_SWE2020
		2009, 2020, 2021	United Kingdom	Bat coronaviruses: M.daubentonii/UK/Wyt/962A<br>M.daubentonii/UK/Wyt/964Co<br>M.daubentonii/UK/Wyt/971Co<br>M.daubentonii/UK/Wyt/972A

				M.daubentonii/UK/Wyt/991C<br>Pedacovirus sp. MdGB01 4-126A<br>Pedacovirus sp. MdGB02 Sample-30<br>Pedacovirus sp. MdGB03 Sample-37<br>Pedacovirus sp. PpiGB01 Sample-25
Myotisemarginatus	Feces, mix=intestinal samples from carcasses of bats	2013, 2015, 2017	France	Alphacoronavirus sp. CS130613<br>Alphacoronavirus sp. FRA_EPI3_3954_1D_p31<br>Alphacoronavirus sp. FRA_EPI3_4019_9Eb_p31<br>Alphacoronavirus sp. FRA_EPI3_4023_10Ab_p31<br>Bat alphacoronavirus Swarming2017_FT201F
		2014	Georgia	Decacovirus sp. GE_CoV10_Myo_ema<br>Sarbecovirus sp. GE_CoV5_Myo_ema
	Feces	2015	Luxembourg	Bat coronavirus BtCoV/Myotis emarginatus/LUX/LUX15_A_272/2015<br>Bat coronavirus BtCoV/Myotis emarginatus/LUX/LUX15_A_276/2015<br>Bat coronavirus BtCoV/Myotis emarginatus/LUX/LUX15_A_284/2015<br>Bat coronavirus BtCoV/Myotis emarginatus/LUX/LUX15_A_293/2015<br>Bat coronavirus BtCoV/Myotis emarginatus/LUX/LUX15_A_301/2015<br>Bat coronavirus BtCoV/Myotis emarginatus/LUX/LUX15_A_46/2015<br>Bat coronavirus BtCoV/Myotis emarginatus/LUX/LUX15_A_48/2015<br>Bat coronavirus BtCoV/Myotis emarginatus/LUX/LUX15_A_55/2015<br>Bat coronavirus BtCoV/Myotis emarginatus/LUX/LUX15_A_59/2015<br>Bat coronavirus BtCoV/Myotis emarginatus/LUX/LUX15_A_63/2015<br>Betacoronavirus 1 BtCoV/Myotis emarginatus/LUX/LUX15_A_158/2015<br>Betacoronavirus 1 BtCoV/Myotis emarginatus/LUX/LUX15_A_288/2015<br>Bat coronavirus BtCoV/Myotis emarginatus/LUX/LUX16_A_1056/2016<br>Bat coronavirus BtCoV/Myotis emarginatus/LUX/LUX16_A_1062/2016<br>Bat coronavirus BtCoV/Myotis emarginatus/LUX/LUX16_A_1079/2016<br>Bat coronavirus BtCoV/Myotis emarginatus/LUX/LUX16_A_1080/2016

				Bat coronavirus BtCoV/Myotis emarginatus/LUX/LUX16_A_1082/2016<br>Bat coronavirus BtCoV/Myotis emarginatus/LUX/LUX16_A_123/2016<br>Bat coronavirus BtCoV/Myotis emarginatus/LUX/LUX16_A_1371/2016<br>Bat coronavirus BtCoV/Myotis emarginatus/LUX/LUX16_A_234/2016<br>Betacoronavirus 1 BtCoV/Myotis emarginatus/LUX/LUX16_A_192/2016<br>Betacoronavirus 1 BtCoV/Myotis emarginatus/LUX/LUX16_A_736/2016
	Swabs	2020	Spain	Bat alphacoronavirus BT97
Myotismyotis	Feces	2017, 2018, 2019	France	Bat alphacoronavirus Swarming2017_FT248F<br>Bat alphacoronavirus PLT_T10B2_Aug<br>Bat alphacoronavirus PLT_T10B2_Mai<br>Bat alphacoronavirus PLT_T1B2_Mai<br>Bat alphacoronavirus PLT_T2B2_Mai<br>Bat alphacoronavirus PLT_T6B2_Mai<br>Bat alphacoronavirus PLT_T9B2_Aug<br>Bat alphacoronavirus Swarming2018_FT18.110F<br>Bat alphacoronavirus Swarming2018_FT18.270F
		2008	Germany	Bat coronavirus N78-10/Germany/2008
		2013	Hungary	CoronavirusBtCoV/B85/HUN/2013
		2016	Italy	Bat coronavirus Mmyo4658_IT_16<br>Bat coronavirus Mmyo4663_IT_16
		2022	Portugal	Bat coronavirus B51<br>Bat coronavirus F15<br>Bat coronavirus F5<br>Bat coronavirus F52
		2007, 2011, 2015	Spain	Alphacoronavirus M.myo/I/Spain/2007<br>Alphacoronavirus sp. FRA_EPI5_Myomyo.1084_D3_<br>Alphacoronavirus sp. FRA_EPI5_Myomyo.936_C2_<br>Alphacoronavirus sp. FRA_EPI9_Myomyo31_4B_P1<br>Alphacoronavirus sp. SPA_EPI2_Myomyo30_2F_<br>Alphacoronavirus sp. SPA_EPI3_4713_1C_p17<br>Alphacoronavirus sp. SPA_EPI3_4714_1D_p17<br>Alphacoronavirus sp. SPA_EPI3_4715_1E_p17<br>Alphacoronavirus sp.

				SPA_EPI5_Myomyo.Minsch52_7D_p25<br>Alphacoronavirus sp.<br>SPA_EPI5_Myomyo25_4A_p25<br>Alphacoronavirus sp.<br>SPA_EPI5_Myomyo_Minsch27_4C_p25<br>Alphacoronavirus sp.<br>SPA_EPI5_Myomyo_Minsch60_8D_p25<br>Alphacoronavirus sp.<br>SPA_EPI5_Myomyo_Minsch61_8E_p25<br>Alphacoronavirus sp.<br>SPA_EPI5_Myomyo_Minsch62_8F_p25<br>Alphacoronavirus sp.<br>SPA_EPI5_Myomyo_Minsch74_10B_p25<br>Alphacoronavirus sp.<br>SPA_EPI7_Myomyo50_7B_p25<br>Alphacoronavirus sp.<br>SPA_EPI7_Myomyo80_10H_p25<br>Alphacoronavirus sp.<br>SPA_EPI9_Myomyo32.1042_4C_P1
		2019, 2021	Switzer-land	Bat alphacoronaviruses:<br>BatCoV/M.myotis/Switzerland/2019<br>BatCoV/M.myotis/Switzerland/2021
		2014	Denmark	Bat coronavirus BtCoV/13585-6/M.nat/DK/2014
	Feces, mix=intestinal samples from carcasses of bats	2013, 2015	France	Alphacoronavirus sp. CS130938<br>Alphacoronavirus sp.<br>FRA_EPI4_Myonat22.4674_8D_P5<br>Alphacoronavirus sp.<br>FRA_EPI4_Myonat24.4677_8G_P5
	Feces	2014	Germany	Bat coronavirus M.nat/GER/3856/2014 188<br>Bat coronavirus M.nat/GER/533A/2014 185<br>Bat coronavirus M.nat/GER/F0BF/2014 165
		2013	Hungary	Coronavirus BtCoV/M67/HUN/2013
Myotisnattereri	Feces, urine	2013, 2014	Italy	Bat coronavirus Mnat1021_IT_14<br>Bat coronavirus Mnat560_IT_13<br>Bat coronavirus Mnat562_IT_13
	Feces	2009	United Kingdom	Bat coronavirus M.nattereri/UK/Sav/1088A<br>Bat coronavirus M.nattereri/UK/Sav/1089B<br>Bat coronavirus M.nattereri/UK/Sav/1091C<br>Bat coronavirus M.nattereri/UK/Sav/1092B<br>Bat coronavirus M.nattereri/UK/Sav/1095A<br>Bat coronavirus M.nattereri/UK/Sav/1106A<br>Bat coronavirus M.nattereri/UK/Sav/1108A<br>Bat coronavirus M.nattereri/UK/Wyt/1011A<br>Bat coronavirus M.nattereri/UK/Wyt/1012B<br>Bat coronavirus M.nattereri/UK/Wyt/1018A<br>Bat coronavirus M.nattereri/UK/Wyt/1019A

				Bat coronavirus M.nattereri/UK/Wyt/1020A<br>Bat coronavirus M.nattereri/UK/Wyt/1023A
Myotisoxygnathus	Feces	2016	Italy	Bat coronavirus Moxy4235_IT_16
Myotispetax		2020	Russia	Bat coronavirus BtCoV/631/RUS/2020
Nyctaluslasiopterus	Feces	2007	Spain	Alphacoronavirus N.las/C/Spain/2007<br>Alphacoronavirus N.las/D/Spain/2007<br>Alphacoronavirus N.las/E/Spain/2007<br>Alphacoronavirus N.las/F/Spain/2007<br>Alphacoronavirus N.las/G/Spain/2007
Nyctalusnoctula	Feces	2010	Italy	BetacoronavirusNyctalusnoctula/Italy/206679-5/2010
		2007, 2008	Netherlands	CoronavirusN.noc/VM176/2007/NLD<br>CoronavirusN.noc/VM182/2007/NLD<br>CoronavirusN.noc/VM199/2007/NLD<br>CoronavirusN.noc/VM366/2008/NLD
		2021	Russia	Bat coronavirus Bat-CoV/N.noct/RU/MOW21-110/1
Pipistrellus	Mix=intestinal samples from carcasses of bats	2013	France	Alphacoronavirus sp. CS130412
		2010	Italy	Betacoronavirus Pipistrellus/Italy/49967-19/2010
	Feces	2007	Spain	Alphacoronavirus P.sp/K/Spain/2007
Pipistrelluskuhlii	Feces, tissue, mix = faecal and organ samples	2010, 2011, 2012, 2014, 2015, 2018, 2021	Italy	Alphacoronavirus Bat-CoV/P.kuhlii/Italy/206645-41/2011<br>Alphacoronavirus Bat-CoV/P.kuhlii/Italy/206679-3/2010<br>Alphacoronavirus Bat-CoV/P.kuhlii/Italy/3398-19/2015<br>Alphacoronavirus Pipistrelluskuhlii/Italy/206645-41/2010<br>Alphacoronavirus Pipistrelluskuhlii/Italy/206679-3/2010<br>Alphacoronavirus sp. BatCoV/19RS495-11/Pipistrelluskuhlii/Italy/2018<br>Bat coronavirus Pkuh605_IT_14<br>BetacoronavirusPipistrelluskuhlii/Italy/206645-27/2011<br>BetacoronavirusPipistrelluskuhlii/Italy/206645-29/2011<br>BetacoronavirusPipistrelluskuhlii/Italy/206645-3/2011<br>BetacoronavirusPipistrelluskuhlii/Italy/206645-53/2011<br>BetacoronavirusPipistrelluskuhlii/Italy/206645-54/2011<br>BetacoronavirusPipistrelluskuhlii/Italy/206645-63/2011

				BetacoronavirusPipistrelluskuhlii/Italy/330375-15/2012<br>Middle East respiratory syndrome coronavirus Bat-CoV/P.khulii/Italy/206645-63/2011<br>Bat alphacoronavirus Bat-CoV/P.kuhlii/Italy/IZSVE_123662/2021<br>Orthocoronavirinae sp. 297348-32<br>Orthocoronavirinae sp. 299160
	Feces	2021	Russia	Bat coronavirus Bat-CoV/P.kuh/RU/ROV21-131/1<br>Bat coronavirus Bat-CoV/P.kuh/RU/ROV21-132/1<br>Bat coronavirus Bat-CoV/P.kuh/RU/ROV21-132/2<br>Bat coronavirus Bat-CoV/P.kuhlii/RU/ROV21-132/3-Nyct<br>Bat coronavirus Bat-CoV/P.kuhlii/RU/ROV21-132/4-Ped
		2007	Spain	Alphacoronavirus P.kuh/Iprima/Spain/2007
Pipistrellusnathusii	Feces	2007, 2012	Germany	Bat coronavirus P.nat/GER/1048/2013 5442<br>Bat coronavirus P.nat/Germany/D5.16/2007<br>Bat coronavirus P.nat/Germany/D5.73/2007
	Feces, tissue, swabs	2017, 2018, 2019, 2020, 2021	Netherlands	Alphacoronavirus sp. Alpha-CoV/P.nathusii/NL/2018-403.3<br>Bat coronavirus BtCoV/P.nathusii/NL/2017-02<br>Bat coronavirus BtCoV/P.nathusii/NL/2017-19<br>Bat coronavirus BtCoV/P.nathusii/NL/2018-02<br>Bat coronavirus BtCoV/P.nathusii/NL/2018-13<br>Bat coronavirus BtCoV/P.nathusii/NL/2018-26<br>Bat coronavirus BtCoV/P.nathusii/NL/2018-30<br>Bat coronavirus BtCoV/P.nathusii/NL/2018-34<br>Bat coronavirus BtCoV/P.nathusii/NL/2018-39<br>Betacoronavirus sp. BtCoV/P.nathusii/NL/2018-403.3<br>Betacoronavirus sp. BtCoV/P.nathusii/NL/2018-413.5<br>Bat coronavirus BtCoV/P.nathusii/NL/2019-01<br>Bat coronavirus BtCoV/P.nathusii/NL/2019-03

				Bat coronavirus BtCoV/P.nathusii/NL/2020-02<br>Bat coronavirus BtCoV/P.nathusii/NL/2021-01<br>Bat coronavirus BtCoV/P.nathusii/NL/2021-02<br>Bat coronavirus BtCoV/P.nathusii/NL/2021-03<br>Bat coronavirus BtCoV/P.nathusii/NL/2021-05
	Feces	2009	Romania	Bat coronavirus BtCoV/8-691/Pip_nat/ROU/2009
		2015	Russia	Middle East respiratory syndrome coronavirus Bat-CoV/P.nathusii/Russia/MOW15-16/1/15<br>Middle East respiratory syndrome coronavirus Bat-CoV/P.nathusii/Russia/MOW15-22/2015<br>Middle East respiratory syndrome coronavirus Bat-CoV/P.nathusii/Russia/MOW15-33/1/15<br>Bat coronavirus Bat-CoV/P.nathusii/Russia/MOW15-16/2<br>Bat coronavirus Bat-CoV/P.nathusii/Russia/MOW15-21/1<br>Bat coronavirus Bat-CoV/P.nathusii/Russia/MOW15-21/2015<br>Bat coronavirus Bat-CoV/P.nathusii/Russia/MOW15-23/1<br>Bat coronavirus Bat-CoV/P.nathusii/Russia/MOW15-23/2015<br>Bat coronavirus Bat-CoV/P.nathusii/Russia/MOW15-33/2
		2011	Ukraine	Bat coronavirus BtCoV/UKR-G17/Pip_nat/UKR/2011
Pipistrelluspipistrellus	Feces, mix=intestinal samples from carcasses of bats	2013, 2014	France	Alphacoronavirus sp. CS130459<br>Alphacoronavirus sp. CS130747<br>Alphacoronavirus sp. CS130786<br>Alphacoronavirus sp. CS130860<br>Alphacoronavirus sp. CS130894<br>Alphacoronavirus Pip1_Cr_FR_2014<br>Alphacoronavirus Pip2_Cr_FR_2014<br>Alphacoronavirus Pip3_M_FR_2014
	Feces, tissue, urine	2014, 2020, 2021	Italy	Bat coronavirus Ppip1000_IT_14<br>Bat coronavirus Ppip1015C_IT_14<br>Bat coronavirus Ppip1016_IT_14<br>Bat coronavirus 60649_2020_(5114)<br>Bat coronavirus 60649_2020_(7243)

				Bat alphacoronavirus 77843_2021<br>Bat alphacoronavirus 77850_2021
	Feces	2008	Nether-lands	CoronavirusP.pipi/VM312/2008/NLD<br>CoronavirusP.pipi/VM314/2008/NLD
Pipistrelluspygmaeus	Feces	2013, 2014, 2015	Denmark	Bat coronavirus BtCoV/B40-5/P.pyg/DK/2013<br>Bat coronavirus BtCoV/7542-55/P.pyg/DK/2014<br>Bat coronavirus BtCoV/OV26-1/P.pyg/DK/2015<br>Bat coronavirus BtCoV/OV27-7/P.pyg/DK/2015<br>Bat coronavirus BtCoV/OV28-11/P.pyg/DK/2015<br>Bat coronavirus BtCoV/OV29-12/P.pyg/DK/2015
		2007, 2013	Germany	Bat coronavirus P.pyg/Germany/D5.70/2007<br>Bat coronavirus P.pyg/Germany/D5.71/2007<br>Bat coronavirus P.pyg/Germany/D5.85/2007<br>Bat coronavirus P.pyg/GER/9848/2014 2830
		2013	Hungary	CoronavirusBtCoV/BS49/HUN/2013
		2009	Romania	Bat coronavirus BtCoV/8-724/Pip_pyg/ROU/2009
		2020	Sweden	Bat coronavirus BtCoV/F-MV1/P.pyg/SWE/2020<br>Bat coronavirus BtCoV/F-MV2/P.pyg/SWE/2020<br>Bat coronavirus BtCoV/F-HS1/P.pyg/SWE/2020
Plecotusauritus	Feces, tissue, mix	2016, 2021	Italy	Bat coronavirus Paur4241_IT_16<br>Orthocoronavirinae sp. 297348-10-int<br>Orthocoronavirinae sp. 297348-10-vis
	Feces	2020	United Kingdom	Merbecovirus sp. PaGB01 5-129B
Rhinolophusblasii	Feces	2008	Bulgaria	Bat coronavirus BB98-15/BGR/2008<br>Bat coronavirus BB98-16/BGR/2008<br>Bat coronavirus BB98-18/BGR/2008<br>Bat coronavirus BB98-41/BGR/2008<br>Bat coronavirus BM48-31/BGR/2008<br>Bat coronavirus BM48-39/BGR/2008<br>Bat coronavirus BM48-48/BGR/2008<br>Bat coronavirus BM98-05/BGR/2008<br>Bat coronavirus BM98-65/BGR/2008<br>Bat coronavirus BR98-12/BGR/2008<br>BetacoronavirusBtCoV/Rhi_bla/BB98-22/BGR/2008<br>BtCoV/Rhi_bla/BB89-22/BGR/2008
Rhinolophuseuryale	Feces	2008, 2009	Bulgaria	Bat coronavirus 1B BR98-19/BGR/2008<br>Bat coronavirus 2B BR98-19/BGR/2008

				Bat coronavirus BB98-43/BGR/2008<br>Bat coronavirus BM48-12/BGR/2008<br>Bat coronavirus BM98-01/BGR/2008<br>Bat coronavirus BM98-05/BGR/2008<br>Bat coronavirus BM98-13/BGR/2008<br>BetacoronavirusBtCoV/Rhi_eur/BB98-92/BGR/2008<br>BtCoV/Rhi_eur/BB89-92/BGR/2008<br>BetacoronavirusBtCoV/Rhi_eur/BB98-98/BGR/2008<br>BtCoV/Rhi_eur/BB89-98/BGR/2008<br>BetacoronavirusBtCoV/Rhi_eur/BB99-04/BGR/2009
		2014	Georgia	Colacovirus sp. GE_CoV71_Rhi_eur<br>Decacovirus sp. GE_CoV152_Rhi_eur<br>Decacovirus sp. GE_CoV153_Rhi_eur<br>Myotacovirus sp. GE_CoV88_Rhi_eur<br>Sarbecovirus sp. GE_CoV166_Rhi_eur<br>Sarbecovirus sp. GE_CoV169_Rhi_eur<br>Sarbecovirus sp. GE_CoV173_Rhi_eur<br>Sarbecovirus sp. GE_Cov174_Rhi_eur<br>Sarbecovirus sp. GE_CoV91_Rhi_eur<br>Sarbecovirus sp. GE_CoV92_Rhi_eur<br>Sarbecovirus sp. GE_Cov93_Rhi_eur<br>Sarbecovirus sp. GE_CoV96_Rhi_eur<br>Sarbecovirus sp. GE_CoV97_Rhi_eur<br>Sarbecovirus sp. GE_CoV98_Rhi_eur
	Feces	2013	Hungary	CoronavirusBtCoV/E63/HUN/2013
Rhinolophusfer-rumequinum	Feces	2008	Bulgaria	Bat coronavirus BM48-28/BGR/2008<br>Bat coronavirus BM48-34/BGR/2008<br>Bat coronavirus BM48-35/BGR/2008<br>Bat coronavirus BNM98-29/BGR/2008
		2011, 2014, 2015, 2018	France	BetacoronavirusBtCoV/Rhi_fer/FR0711-B11/FRA/2011<br>BetacoronavirusBtCoV/Rhi_fer/FR0711-B3/FRA/2011<br>Alphacoronavirus sp. FRA_EPI4_Rhfer6_F1_P10<br>Alphacoronavirus sp. FRA_EPI6_Rhfer.Myema.pool26_D11_P6<br>Alphacoronavirus sp. FRA_EPI6_Rhfer.Myema.pool28_F5_P6<br>Alphacoronavirus sp. FRA_EPI6_Rhfer1004_5H_P23<br>Alphacoronavirus sp. FRA_EPI6_Rhfer1006_6B_P23<br>Alphacoronavirus sp. FRA_EPI6_Rhfer965_1A_P23

Alphacoronavirus sp.
FRA_EPI6_Rhfer967_1C_P23
Alphacoronavirus sp.
FRA_EPI6_Rhfer976_2D_P23
Alphacoronavirus sp.
FRA_EPI6_Rhfer980_2H_P23
Alphacoronavirus sp.
FRA_EPI6_Rhfer982_3B_P23
Alphacoronavirus sp.
FRA_EPI6_Rhfer984_3D_P23
Alphacoronavirus sp.
FRA_EPI6_Rhfer986_3F_P23
Alphacoronavirus sp.
FRA_EPI6_Rhfer987_3G_P23
Alphacoronavirus sp.
FRA_EPI6_Rhfer996_4H_P23
Alphacoronavirus sp.
FRA_EPI6_Rhfer997_5A_P23
Alphacoronavirus sp.
FRA_EPI6_Rhfer999_5C_P23
Alphacoronavirus sp.
Betacoronavirus sp.
FRA_EPI1_Rhfer.pool1_B11_P10
Betacoronavirus sp.
FRA_EPI1_Rhfer17_A3_P10
Betacoronavirus sp.
FRA_EPI1_Rhfer19_C3_P10
Betacoronavirus sp.
FRA_EPI1_Rhfer1_A1_P10
Betacoronavirus sp.
FRA_EPI1_Rhfer23_G3_P10
Betacoronavirus sp.
FRA_EPI1_Rhfer33_A5_P10
Betacoronavirus sp.
FRA_EPI1_Rhfer39_G5_P10
Betacoronavirus sp.
FRA_EPI1_Rhfer42_B6_P10
Betacoronavirus sp.
FRA_EPI1_Rhfer46_3E_P29
Betacoronavirus sp.
FRA_EPI1_Rhfer47_G6_P10
Betacoronavirus sp.
FRA_EPI1_Rhfer57_A8_P10
FRA_EPI2_Rhfer.3970_3D_p31
Betacoronavirus sp. FRA_EPI1_3870_2H_P30
Betacoronavirus sp. FRA_EPI1_3871_3A_P30
Betacoronavirus sp. FRA_EPI1_3874_3D_P30
Betacoronavirus sp. FRA_EPI1_3875_3E_P30

Betacoronavirus sp. FRA_EPI1_3877_3G_P30
Betacoronavirus sp. FRA_EPI1_3879_4A_P30
Betacoronavirus sp. FRA_EPI1_3880_4B_P30
Betacoronavirus sp. FRA_EPI1_3881_4C_P30
Betacoronavirus sp. FRA_EPI1_3882_4D_P30
Betacoronavirus sp. FRA_EPI1_3889_5C_P30
Betacoronavirus sp. FRA_EPI1_3894_5H_P30
Betacoronavirus sp. FRA_EPI1_3897_6C_P30
Betacoronavirus sp. FRA_EPI1_3899_6E_P30
Betacoronavirus sp. FRA_EPI1_3900_6F_P30
Betacoronavirus sp. FRA_EPI1_3921_9C_P30
Betacoronavirus sp. FRA_EPI1_3923_9E_P30
Betacoronavirus sp. FRA_EPI1_3924_9F_P30
Betacoronavirus sp. FRA_EPI1_3925_9G_P30
Betacoronavirus sp. FRA_EPI1_3928_10B_P30
Betacoronavirus sp. FRA_EPI1_3930_10D_P30
Betacoronavirus sp. FRA_EPI1_3932_10F_P30
Betacoronavirus sp. FRA_EPI1_3940_11F_P30
Betacoronavirus sp. FRA_EPI1_3944_12B_P30
Betacoronavirus sp. FRA_EPI1_3952_1B_p31
Betacoronavirus sp. FRA_EPI1_3956_1F_p31
Betacoronavirus sp. FRA_EPI1_3957_1G_p31
Betacoronavirus sp. FRA_EPI1_3958_1H_p31
Betacoronavirus sp. FRA_EPI1_3959_2A_p31
Betacoronavirus sp. FRA_EPI1_3965_2G_p31
Betacoronavirus sp. FRA_EPI1_3966_2H_p31
Betacoronavirus sp. FRA_EPI1_3968_3B_p31
Betacoronavirus sp. FRA_EPI1_3971_3E_p31
Betacoronavirus sp. FRA_EPI1_3975_4A_p31
Betacoronavirus sp. FRA_EPI1_3978_4D_p31
Betacoronavirus sp. FRA_EPI1_4000_7B_p31
Betacoronavirus sp. FRA_EPI1_4005_7G_p31
Betacoronavirus sp. FRA_EPI1_4006_7H_p31
Betacoronavirus sp. FRA_EPI1_4015_9A_p31
Betacoronavirus sp. FRA_EPI1_4016_9B_p31
Betacoronavirus sp. FRA_EPI1_4019_9E_p31
Betacoronavirus sp. FRA_EPI1_4023_10A_p31
Betacoronavirus sp. FRA_EPI1_Rhfer59_11B_P29
Betacoronavirus sp. FRA_EPI1_Rhfer59_5B_P29
Betacoronavirus sp.

				FRA_EPI1_Rhfer60_5C_P29<br>Betacoronavirus sp.<br>FRA_EPI1_Rhfer61_E8_P10<br>Betacoronavirus sp.<br>FRA_EPI1_Rhfer63_G8_P10<br>Betacoronavirus sp.<br>FRA_EPI1_Rhfer66_6A_P29<br>Betacoronavirus sp.<br>FRA_EPI1_Rhfer67_6B_P29<br>Betacoronavirus sp.<br>FRA_EPI1_Rhfer975_2C_P23<br>Bat alphacoronavirus Swarm-ing2018_FT18.164F
Rhinolophusfer-rumequinumc.d	Feces	2014	Georgia	Myotacovirus sp. GE_CoV58_Rhi_fer<br>Sarbecovirus sp. GE_CoV11_Rhi_fer<br>Sarbecovirus sp. GE_CoV13_Rhi_fer<br>Sarbecovirus sp. GE_CoV17_Rhi_fer<br>Sarbecovirus sp. GE_CoV18_Rhi_fer<br>Sarbecovirus sp. GE_CoV19_Rhi_fer<br>Sarbecovirus sp. GE_CoV21_Rhi_fer
		2013	Hungary	CoronavirusBtCoV/M8/HUN/2013
		2009, 2016	Italy	Coronavirus SarBatCoV1<br>BetacoronavirusBtCoV/Rhi_fer/It1/ITA/2009<br>BetacoronavirusBtCoV/Rhi_fer/It13/ITA/2009<br>BetacoronavirusBtCoV/Rhi_fer/It15/ITA/2009<br>BetacoronavirusBtCoV/Rhi_fer/It17/ITA/2009<br>BetacoronavirusBtCoV/Rhi_fer/It2/ITA/2009<br>Bat coronavirus Rfer4009_IT_16<br>Bat coronavirus Rfer4011_IT_16<br>Bat coronavirus Rfer4015_IT_16<br>Bat coronavirus Rfer4019_IT_16<br>Bat coronavirus Rfer4024_IT_16<br>Bat coronavirus Rfer4025_IT_16<br>Bat coronavirus Rfer4027_IT_16<br>Bat coronavirus Rfer4674_IT_16<br>Bat coronavirus Rfer4675_IT_2016
		2016	Luxem-bourg	Severe acute respiratory syndrome-related coronavirus BtCoV/Rhinolophus ferrumequi-num/LUX/LUX16_A_24/2016<br>Severe acute respiratory syndrome-related coronavirus BtCoV/Rhinolophus ferrumequi-num/LUX/LUX16_A_37/2016
		2022	Portugal	Bat coronavirus F30
		2020	Russia	Bat SARS-like coronavirus Khosta-1<br>BtCoV/Khosta-1/Rh/Russia/2020
		2015	Spain	Alphacoronavirus sp.<br>SPA_EPI7_Rhifer1087_10E_p25<br>Betacoronavirus sp.

				SPA_EPI1_Rhfer13_11D_P24 Betacoronavirus sp. SPA_EPI1_Rhfer15_11F_P24 Betacoronavirus sp. SPA_EPI1_Rhfer19_12B_P24 Betacoronavirus sp. SPA_EPI1_Rhfer25_6H_P24
		2021	United Kingdom	Sarbecovirus sp. RfGB01 1-GH087 Sarbecovirus sp. RfGB02 2-GH106
Rhinolophushippo-sideros	Feces, mix	2013	Hungary	CoronavirusBtCoV/M9/HUN/2013
		2011, 2012, 2020, 2021	Italy	BetacoronavirusRhinolophushipposideros/Italy/196814/2011 BetacoronavirusRhinolophushipposideros/Italy/187632-2/2012 BetacoronavirusRhinolophushipposideros/Italy/243585/2012 Orthocoronavirinae sp. 297164 Orthocoronavirinae sp. 212520-3 Orthocoronavirinae sp. 245878 Orthocoronavirinae sp. 308728
		2021, 2022	Poland	SARS-related betacoronavirus BtCoV_96 SARS-related betacoronavirus BtCoV_26 SARS-related betacoronavirus BtCoV_28
	Feces	2020	Russia	Bat SARS-like coronavirus Khosta-2 BtCoV/Khosta-2/Rh/Russia/2020
		2008, 2009	Slovenia	Bat coronavirus SLO1A0050/2008/SVN Bat coronavirus SLO1A0066/2008/SVN Bat coronavirus SLO1A0082/2008/SVN Betacoronaviruses: BtCoV/Rhi_hip/Slo48/SLO/2009 BtCoV/Rhi_hip/Slo52/SLO/2009 BtCoV/Rhi_hip/Slo53/SLO/2009 BtCoV/Rhi_hip/Slo54/SLO/2009 BtCoV/Rhi_hip/Slo57/SLO/2009 BtCoV/Rhi_hip/Slo69/SLO/2009
		2010	Spain	BetacoronavirusBtCoV/Rhi_hip/R07-09/SPA/2010 BetacoronavirusBtCoV/Rhi_hip/R13-08/SPA/2010 BetacoronavirusBtCoV/Rhi_hip/R46-03/SPA/2010 BetacoronavirusBtCoV/Rhi_hip/R7-08/SPA/2010 BetacoronavirusBtCoV/Rhi_hip/R77-02/SPA/2010 BetacoronavirusBtCoV/Rhi_hip/R8-09/SPA/2010
		2020, 2021	United Kingdom	Sarbecovirus RhGB01 Sarbecovirus sp. RhGB07 2-30B

				Sarbecovirus sp. RhGB08 Sample-18<br>Sarbecovirus sp. RhGB02<br>Sarbecovirus sp. RhGB05<br>Sarbecovirus sp. RhGB06
Rhinolophusmehelyi	Feces	2008	Bulgaria	Bat coronavirus BM48-32/BGR/2008<br>Bat coronavirus BM98-07/BGR/2008
		2022	Portugal	Bat coronavirus F11
Vespertiliomurinus	Feces	2021	Russia	Bat coronavirus Bat-CoV/V.mur/RU/MOW21-186/1
		2019, 2020, 2021	Switzer-land	Middle East respiratory syndrome corona-virus BatCoV/V.murinus/Switzerland/2019<br>Middle East respiratory syndrome corona-virus BatCoV/V.murinus/Switzerland/2020<br>Middle East respiratory syndrome corona-virus BatCoV/V.murinus/Switzerland/2021
unclassifiedChiroptera	Feces	2020	Croatia	Bat coronaviruses BD10, BD8, BS2
		2014	Finland	Bat alphacoronaviruses:<br>BtCoV/347_14/FIN/2014<br>BtCoV/417_14/FIN/2014
	Tissue		Germany	Bat coronavirus 210/09 P
	Feces	2022	Italy	Orthocoronavirinae sp. 221166<br>Orthocoronavirinae sp. 277844
