# Supplementary material for: Bat-Borne Viruses and Pandemic Risk: Could Europe Be an Emergence Hotspot?
Source: Viruses. 2026 May 2;18(5):535. doi: 10.3390/v18050535 (PMC13211416; doi:10.3390/v18050535)
Supplement: Supplementary file 1 [file viruses-18-00535-s001.zip › Skowron et al. - Table S2.pdf]

Supplementary Table S2. Cases of Rhabdovirus isolation from bats in Europe.

Bat species	Sample type	Collection year	Sampling country	Viruses
Eptesicusisabellinus	Swabs, tissue	1999, 2002, 2006, 2007, 2008, 2009, 2011	Spain	European bat 1 lyssavirus 155R99 Eptesicusisabellinus rhabdovirus 4 Spain.3x24636.02<br>Eptesicusisabellinus rhabdovirus 1 Spain.3x30464.06<br>Eptesicusisabellinus rhabdovirus 2 Spain.3x30632.07<br>Eptesicusisabellinus rhabdovirus 3 Spain.3x31119.07<br>Eptesicusisabellinus rhabdovirus 5 Spain.3x24636.07<br>European bat 1 lyssavirus 211R07 European bat 1 lyssavirus 292R07 European bat 1 lyssavirus RV2416 European bat 1 lyssavirus 86R08<br>European bat 1 lyssavirus 28458<br>European bat lyssavirus 201127004SP European bat lyssavirus 201149008SP
Eptesicusnilssoni	Swabs		Germany	Rhabdovirus 26873<br>Rhabdovirus 26876
Eptesicusserotinus	Culture, tissue	1985, 1986, 1987, 1993, 1995, 1997, 1999, 2000, 2002	Denmark	European bat 1 lyssavirus 02016DEN<br>European bat 1 lyssavirus BATDANMARK<br>European bat 1 lyssavirus EBLV-1a-Duv07<br>European bat 1 lyssavirus G02007.DEN_1<br>European bat 1 lyssavirus G02010.DEN_1<br>European bat 1 lyssavirus G02011.DEN_1<br>European bat 1 lyssavirus G02013.DEN_1<br>European bat 1 lyssavirus G94109.DEN_1<br>European bat 1 lyssavirus G9479.DEN_1<br>European bat 1 lyssavirus RV20<br>European bat lyssavirus 02007.DEN<br>European bat lyssavirus 02010.DEN<br>European bat lyssavirus 02011.DEN<br>European bat lyssavirus 02012.DEN<br>European bat lyssavirus 02013.DEN<br>European bat lyssavirus 02015.DEN<br>European bat lyssavirus 94109.DEN<br>European bat lyssavirus 94110.DEN<br>European bat lyssavirus 9479.DEN<br>European bat lyssavirus G02007.DEN_2<br>European bat lyssavirus G02010.DEN_2

			European bat lyssavirus G02011.DEN_2<br>European bat lyssavirus G02013.DEN_2<br>European bat lyssavirus G94109.DEN_2<br>European bat lyssavirus G9479.DEN_2
Culture, tissue	1985, 1989, 1995, 1997, 1998, 1999, 2000, 2001, 2002, 2003, 2004, 2005, 2006, 2007, 2008, 2009, 2010, 2011, 2012, 2014, 2015	France	European bat 1 lyssavirus 00002FRA<br>European bat 1 lyssavirus 00003FRA<br>European bat 1 lyssavirus 00132<br>European bat 1 lyssavirus 02031FRA<br>European bat 1 lyssavirus 02032FRA<br>European bat 1 lyssavirus 02033FRA<br>European bat 1 lyssavirus 03002FRA<br>European bat 1 lyssavirus 04032FRA<br>European bat 1 lyssavirus 05001FRA<br>European bat 1 lyssavirus 05002FRA<br>European bat 1 lyssavirus 06001FRA<br>European bat 1 lyssavirus 06002FRA<br>European bat 1 lyssavirus 07057FRA<br>European bat 1 lyssavirus 07058FRA<br>European bat 1 lyssavirus 08120FRA<br>European bat 1 lyssavirus 08341FRA<br>European bat 1 lyssavirus 09034FRA<br>European bat 1 lyssavirus 107251<br>European bat 1 lyssavirus 113852<br>European bat 1 lyssavirus 116883<br>European bat 1 lyssavirus 120914<br>European bat 1 lyssavirus 121411<br>European bat 1 lyssavirus 121633<br>European bat 1 lyssavirus 122154<br>European bat 1 lyssavirus 122276<br>European bat 1 lyssavirus 122319<br>European bat 1 lyssavirus 122938<br>European bat 1 lyssavirus 123008<br>European bat 1 lyssavirus 123801<br>European bat 1 lyssavirus 124193<br>European bat 1 lyssavirus 124345<br>European bat 1 lyssavirus 124489<br>European bat 1 lyssavirus 126235<br>European bat 1 lyssavirus 126669<br>European bat 1 lyssavirus 127051<br>European bat 1 lyssavirus 127834<br>European bat 1 lyssavirus 127835<br>European bat 1 lyssavirus 128210<br>European bat 1 lyssavirus 128633<br>European bat 1 lyssavirus 128635<br>European bat 1 lyssavirus 128636<br>European bat 1 lyssavirus 128637<br>European bat 1 lyssavirus 128665<br>European bat 1 lyssavirus 128681

	European bat 1 lyssavirus 128683
	European bat 1 lyssavirus 128708
	European bat 1 lyssavirus 128827
	European bat 1 lyssavirus 129051
	European bat 1 lyssavirus 129055
	European bat 1 lyssavirus 129087
	European bat 1 lyssavirus 129090
	European bat 1 lyssavirus 129116
	European bat 1 lyssavirus 129123
	European bat 1 lyssavirus 129246
	European bat 1 lyssavirus 129290
	European bat 1 lyssavirus 129394
	European bat 1 lyssavirus 129396
	European bat 1 lyssavirus 129409
	European bat 1 lyssavirus 129428
	European bat 1 lyssavirus 129666
	European bat 1 lyssavirus 129865
	European bat 1 lyssavirus 129944
	European bat 1 lyssavirus 130544
	European bat 1 lyssavirus 130576
	European bat 1 lyssavirus 130662
	European bat 1 lyssavirus 130904
	European bat 1 lyssavirus 131054
	European bat 1 lyssavirus 15007FRA
	European bat 1 lyssavirus 15521
	European bat 1 lyssavirus 78983
	European bat 1 lyssavirus 8918FRA
	European bat 1 lyssavirus 8919FRA
	European bat 1 lyssavirus 9906FRA
	European bat 1 lyssavirus Ancy sur Moselle
	European bat 1 lyssavirus EBL1FRA
	European bat 1 lyssavirus G0001.FRA_1
	European bat 1 lyssavirus G0001.FRA_2
	European bat 1 lyssavirus G0102.FRA_1
	European bat 1 lyssavirus G0102.FRA_2
	European bat 1 lyssavirus G02032.FRA_1
	European bat 1 lyssavirus G02033.FRA_1
	European bat 1 lyssavirus G03002.FRA_1
	European bat 1 lyssavirus G8919.FRA_1
	European bat 1 lyssavirus G9603.FRA_1
	European bat 1 lyssavirus G9603.FRA_2
	European bat lyssavirus 0001.FRA
	European bat lyssavirus 0002.FRA
	European bat lyssavirus 0003.FRA
	European bat lyssavirus 0102.FRA
	European bat lyssavirus 02031.FRA
	European bat lyssavirus 02032.FRA

			European bat lyssavirus 02033.FRA<br>European bat lyssavirus 03002.FRA<br>European bat lyssavirus 8918.FRA<br>European bat lyssavirus 8919.FRA<br>European bat lyssavirus 9603.FRA<br>European bat lyssavirus 9906.FRA<br>European bat lyssavirus G02032.FRA_2<br>European bat lyssavirus G02033.FRA_2<br>European bat lyssavirus G03002.FRA_2<br>European bat lyssavirus G8919.FRA_2<br>European bat lyssavirus G_122938-2002<br>Rabies lyssavirus 128683<br>Rabieslyssavirus 129668
Culture, tissue, swabs	1968, 1970, 1982, 1985, 1986, 1987, 1988, 1989, 1990, 1993, 1996, 1997, 1998, 1999, 2002, 2003, 2004, 2005, 2006, 2007, 2008, 2009, 2010, 2011, 2012	Germany	European bat 1 lyssavirus 10850<br>European bat 1 lyssavirus 11647<br>European bat 1 lyssavirus 15730<br>European bat 1 lyssavirus 16902<br>European bat 1 lyssavirus 16908<br>European bat 1 lyssavirus 18720<br>European bat 1 lyssavirus 18814<br>European bat 1 lyssavirus 18822<br>European bat 1 lyssavirus 19926<br>European bat 1 lyssavirus 20170<br>European bat 1 lyssavirus 20171<br>European bat 1 lyssavirus 20174<br>European bat 1 lyssavirus 2136_GR<br>European bat 1 lyssavirus 21836<br>European bat 1 lyssavirus 23539<br>European bat 1 lyssavirus 23544<br>European bat 1 lyssavirus 24525<br>European bat 1 lyssavirus 24529<br>European bat 1 lyssavirus 24610<br>European bat 1 lyssavirus 24746<br>European bat 1 lyssavirus 24831<br>European bat 1 lyssavirus 24832<br>European bat 1 lyssavirus 25006<br>European bat 1 lyssavirus 25495<br>European bat 1 lyssavirus 25900<br>European bat 1 lyssavirus 27904<br>European bat 1 lyssavirus 28228<br>European bat 1 lyssavirus 30561<br>European bat 1 lyssavirus 30575<br>European bat 1 lyssavirus 31054<br>European bat 1 lyssavirus 31177<br>European bat 1 lyssavirus 31178<br>European bat 1 lyssavirus 3132<br>European bat 1 lyssavirus 3135<br>European bat 1 lyssavirus 31448

European bat 1 lyssavirus 32054
European bat 1 lyssavirus 32062
European bat 1 lyssavirus 4644
European bat 1 lyssavirus 5185
European bat 1 lyssavirus 5248
European bat 1 lyssavirus 5254
European bat 1 lyssavirus 5300
European bat 1 lyssavirus 5304
European bat 1 lyssavirus 7467
European bat 1 lyssavirus 7471
European bat 1 lyssavirus 8215
European bat 1 lyssavirus 8624
European bat 1 lyssavirus 905
European bat 1 lyssavirus 915
European bat 1 lyssavirus 932
European bat 1 lyssavirus 933
European bat 1 lyssavirus 934
European bat 1 lyssavirus 9395GER
European bat 1 lyssavirus 9396GER
European bat 1 lyssavirus 9399GER
European bat 1 lyssavirus 9436GER
European bat 1 lyssavirus 9438GER
European bat 1 lyssavirus 9440GER
European bat 1 lyssavirus 9477GER
European bat 1 lyssavirus 9588
European bat 1 lyssavirus 959
European bat 1 lyssavirus 989
European bat 1 lyssavirus 992
European bat 1 lyssavirus 998
European bat 1 lyssavirus BATSTADE
European bat 1 lyssavirus G9395.GER_1
European bat 1 lyssavirus G9396.GER_1
European bat 1 lyssavirus G9398.GER_1
European bat 1 lyssavirus G9398.GER_2
European bat 1 lyssavirus G9399.GER_1
European bat 1 lyssavirus G94109.DEN_1
European bat 1 lyssavirus G9437.GER_1
European bat 1 lyssavirus G9439.GER_1
European bat 1 lyssavirus G9440.GER_1
European bat 1 lyssavirus G9441.GER_1
European bat 1 lyssavirus G9479.DEN_1
European bat 1 lyssavirus G9481.GER_1
European bat 1 lyssavirus RV1423
European bat 1 lyssavirus RV9
European bat 1 lyssavirus RV9; 9395GER
European bat lyssavirus 9395.GER
European bat lyssavirus 9396.GER
European bat lyssavirus 9398.GER

			European bat lyssavirus 9399.GER<br>European bat lyssavirus 9436.GER<br>European bat lyssavirus 9437.GER<br>European bat lyssavirus 9438.GER<br>European bat lyssavirus 9439.GER<br>European bat lyssavirus 9440.GER<br>European bat lyssavirus 9441.GER<br>European bat lyssavirus 9477.GER<br>European bat lyssavirus 9481.GER<br>European bat lyssavirus G9395.GER_2<br>European bat lyssavirus G9396.GER_2<br>European bat lyssavirus G9399.GER_2<br>European bat lyssavirus G9437.GER_2<br>European bat lyssavirus G9439.GER_2<br>European bat lyssavirus G9440.GER_2<br>European bat lyssavirus G9441.GER_2<br>European bat lyssavirus G9481.GER_2<br>Rhabdovirus 27676
	1999, 2009, 2010, 2011, 2012, 2015	Hungary	European bat 1 lyssavirus Hun_bat_1999_1<br>European bat 1 lyssavirus Hun_bat_2009_1<br>European bat 1 lyssavirus Hun_bat_2010_1<br>European bat 1 lyssavirus Hun_bat_2011_1<br>European bat 1 lyssavirus Hun_bat_2011_2<br>European bat 1 lyssavirus Hun_bat_2012_1<br>European bat 1 lyssavirus Hun_bat_2015<br>European bat lyssavirus 22540<br>European bat lyssavirus 52206
Tissue	2013	Luxembourg	European bat 1 lyssavirus DR707
Culture, tissue	1987, 1989, 1992, 1993, 1997	Netherlands	European bat lyssavirus 9372.HOL<br>European bat lyssavirus 9376.HOL<br>European bat lyssavirus 9377.HOL<br>European bat lyssavirus 94113.HOL<br>European bat lyssavirus 94115.HOL<br>European bat lyssavirus 94116.HOL<br>European bat lyssavirus 9478.HOL<br>European bat lyssavirus 9480.HOL<br>European bat lyssavirus G02024.HOL_2<br>European bat lyssavirus G9366.HOL_2<br>European bat lyssavirus G9367.HOL_2<br>European bat lyssavirus G9372.HOL_2<br>European bat lyssavirus G9376.HOL_2<br>European bat lyssavirus G94113.HOL_2

				European bat lyssavirus G94116.HOL_2<br>European bat lyssavirus G9480.HOL_2<br>Rabieslyssavirus 9480HOL
	Culture, tissue	1985, 1989, 1990, 1994, 1998, 2014	Poland	European bat 1 lyssavirus 001N<br>European bat 1 lyssavirus 019N<br>European bat 1 lyssavirus 021N<br>European bat 1 lyssavirus 022N<br>European bat 1 lyssavirus 8615POL<br>European bat 1 lyssavirus 9394POL<br>European bat 1 lyssavirus EBL1POL<br>European bat 1 lyssavirus G9394.POL_1<br>European bat lyssavirus 8615.POL<br>European bat lyssavirus 9394.POL<br>European bat lyssavirus 96031.POL<br>European bat lyssavirus G9394.POL_2<br>Rabies lyssavirus 019N<br>Rabies lyssavirus 067N
	Culture	1985, 2019	Russia	European bat 1 lyssavirus 9397RUS<br>European bat 1 lyssavirus Vor-1
	Culture, tissue	2001	Slovakia	European bat 1 lyssavirus G01018.FRA_1<br>European bat 1 lyssavirus G01018.FRA_2<br>European bat lyssavirus 01018.SLO<br>European bat 1 lyssavirus 01018SLO
	Culture, tissue	1987, 1994, 2002, 2016	Spain	European bat lyssavirus 94285.SPA<br>European bat lyssavirus 9483.SPA<br>European bat 1 lyssavirus 201640425<br>European bat 1 lyssavirus 44R02<br>European bat 1 lyssavirus 94285ESP<br>European bat 1 lyssavirus 94285SPA<br>European bat 1 lyssavirus 9483SPA<br>European bat 1 lyssavirus g94285_1<br>European bat 1 lyssavirus g94285_2<br>European bat 1 lyssavirus G9483.SPA_1<br>European bat 1 lyssavirus G9483.SPA_2
		2017	Switzerland	European bat 1 lyssavirus TW
	Culture	1987	Ukraine	European bat 1 lyssavirus 9443UKR
Hypsugosavii	Swabs	2007	Spain	Hypsugosaviirhabdovirus 1 Spain.070613.07
Miniopterusschreibersii	Tissue	2017	France	Lleida bat lyssavirus 131989
	Tissue, swabs	2008, 2011, 2012	Spain	Lleida bat lyssavirus lleidabat2012<br>Lleida bat lyssavirus RV3208<br>Mediterranean bat virus 2012096<br>Miniopterusschreibersii rhabdovirus 1 Spain.080623.08
	Tissue	2002	Russia	West Caucasian bat lyssavirus WCBV
Murinaleucogaster	Tissue	2002	Russia	Irkutlyssavirus IRKV
Myotisbrandtii	Tissue	2017	Finland	Kotalahti bat lyssavirus Ra696-2017
Myotiscapaccinii	Tissue	2014	Slovenia	Divaea bat lyssavirus PP-0868

Myotisdasycneme	CultureTissue<br>Tissue<br><br>Tissue<br>Tissue	1898, 1986, 1987, 1989, 1993<br>1993<br>1993<br>1986<br>1987	Netherlands	European bat 2 lyssavirus 92666<br>European bat 2 lyssavirus 9018HOL European bat 2 lyssavirus 9018.HOL<br>European bat 2 lyssavirus 9375.HOL<br>European bat 2 lyssavirus 9375HOL<br>European bat 2 lyssavirus 94112.HOL<br>European bat 2 lyssavirus 94112HOL<br>European bat 2 lyssavirus EBL2HOL<br>European bat 2 lyssavirus 47072
	Swabs	2013	Denmark	European bat 2 lyssavirus EBLV-2_Daubentons_bat_Denmark_2013<br>Rhabdovirus RV-DK1<br>Rhabdovirus RV-DK2
	CultureTissue<br>Mix	2009,<br>2016	Finland	European bat 2 lyssavirus ra3278<br>European bat 2 lyssavirus 2Finland2016<br>European bat 2 lyssavirus 3278_09
	Tissue	2006, 2007, 2012, 2013	Germany	European bat 2 lyssavirus RV2506<br>European bat 2 lyssavirus F500LGL<br>European bat 2 lyssavirus F860LGL<br>European bat 2 lyssavirus GER-16618<br>European bat 2 lyssavirus 16618<br>European bat 2 lyssavirus 18856<br>European bat 2 lyssavirus 25538<br>European bat 2 lyssavirus 31452
		2015	Norway	European bat 2 lyssavirus NO-2015-04-18832<br>European bat 2 lyssavirus 18832
Myotisdaubentonii			Russia	Rabies lyssavirus Bat-BA<br>Rabies lyssavirus Bat-BN1<br>Rabieslyssavirus Bat-YN1
	Tissue<br>Culture<br>Swabs	1992, 1993<br>2002, 2009	Switzerland	European bat 2 lyssavirus 9337.SWI<br>European bat 2 lyssavirus 9337SWI<br>European bat 2 lyssavirus 02054SWI<br>European bat 2 lyssavirus 02055SWI<br>European bat 2 lyssavirus 118_02_mp1<br>European bat 2 lyssavirus 1397_mp3<br>European bat 2 lyssavirus TW0118<br>European bat 2 lyssavirus TW118/02<br>European bat 2 lyssavirus TW1392<br>European bat 2 lyssavirus TW1814<br>European bat 2 lyssavirus TW1814/92<br>European bat 2 lyssavirus 1814_mp2<br>European bat 2 lyssavirus 70B_mp3
	Tissue	1996, 2002, 2003, 2004, 2006, 2007,	United Kingdom	European bat 2 lyssavirus M08/09<br>European bat 2 lyssavirus RV1332<br>European bat 2 lyssavirus RV1787<br>European bat 2 lyssavirus RV1788<br>European bat 2 lyssavirus RV2159

		2008,<br>2009,<br>2014,<br>2015,<br>2016,<br>2017		European bat 2 lyssavirus RV2336<br>European bat 2 lyssavirus RV2418<br>European bat 2 lyssavirus RV2473<br>European bat 2 lyssavirus RV2482<br>European bat 2 lyssavirus RV2974<br>European bat 2 lyssavirus RV3158<br>European bat 2 lyssavirus RV3369<br>European bat 2 lyssavirus RV3370<br>European bat 2 lyssavirus RV3385<br>European bat 2 lyssavirus RV628
Myotismyotis	Blood		Spain	European bat 1 lyssavirus 00-083<br>European bat 1 lyssavirus 00-099<br>European bat 1 lyssavirus 00-103<br>European bat 1 lyssavirus 00-107<br>European bat 1 lyssavirus 00-119<br>European bat 1 lyssavirus 01-076<br>European bat 1 lyssavirus 01-077<br>European bat 1 lyssavirus 02-078<br>European bat 1 lyssavirus 02-085<br>European bat 1 lyssavirus 03-085<br>European bat 1 lyssavirus 03-093<br>European bat 1 lyssavirus 03-097<br>European bat 1 lyssavirus 03-125<br>European bat 1 lyssavirus 03-247<br>European bat 1 lyssavirus 03-254<br>European bat 1 lyssavirus 03-292<br>European bat 1 lyssavirus 03-298<br>European bat 1 lyssavirus 04-121<br>European bat 1 lyssavirus 04-135<br>European bat 1 lyssavirus 04-215<br>European bat 1 lyssavirus 05-409
Myotismystacinus	Swabs		Germany	Rhabdovirus 26855
Myotisnattereri	Swabs<br>Tissue	2010, 2012	Germany	Rhabdovirus 26530<br>Rhabdovirus 26907<br>Bokeloh bat lyssavirus 21961_BBLV<br>Bokeloh bat lyssavirus 29008
		2016	Poland	Bokeloh bat lyssavirus 080N
	Tissue	2012, 2013	France	Bokeloh bat lyssavirus 129700<br>Bokeloh bat lyssavirus 13001FRA
Pipistrelluskuhlii	Culture	2016	Italy	Vapriovirus VAPV_2016
Pipistrellusnathusii	Tissue	1992	Germany	European bat 1 lyssavirus 976
Pipistrelluspipistrellus	Tissue	1994	Germany	European bat 1 lyssavirus 5250
Plecotusauritus	Tissue	1996	Germany	European bat 1 lyssavirus 5226
	Swabs	2008	Spain	Plecotusauritusrhabdovirus 1 Spain.080603.08
			Russia	Rabieslyssavirus Bat-PA
Rhinolophusferrumequi-num	Swabs	2004, 2008	Spain	Rhinolophus ferrumequinum rhabdovirus 1 Spain.3x28660.04

				Rhinolophus ferrumequinum rhabdovirus 2 Spain.080514.08
Rousettusaegyptiacus	Culture	1999	France	Lagos bat lyssavirus 119645
Vespertiliomurinus	Tissue	1987	Ukraine	European bat 1 lyssavirus G9443.UKR_1<br>European bat lyssavirus 9443.UKR<br>European bat lyssavirus G9443.UKR_2
unclassifiedChiroptera	Culture	1993	Denmark	European bat 1 lyssavirus 02007DEN<br>European bat 1 lyssavirus Rv19<br>European bat 1 lyssavirus Rv24
	Tissue		Finland	European bat 2 lyssavirus 9007FIN
	Culture, tissue	2013	France	Bokeloh bat lyssavirus 1301FRA
	Culture,, swabs, tissue	1978, 1986, 1999, 2000, 2001, 2003, 2004	Germany	European bat 1 lyssavirus RV145<br>European bat 1 lyssavirus Bat B24<br>European bat 1 lyssavirus 4895<br>European bat 1 lyssavirus 5006<br>European bat 1 lyssavirus 5009<br>European bat 1 lyssavirus 5665<br>European bat 1 lyssavirus 8482<br>European bat 1 lyssavirus 9394GER European bat 1 lyssavirus 9908<br>European bat 1 lyssavirus Rv11<br>Rhabdovirus 29130
	Culture	1988	Netherlands	European bat 1 lyssavirus RV31<br>European bat 2 lyssavirus Rv228<br>Rabies lyssavirus NLBat13<br>Rabies lyssavirus NLBat15<br>Rabies lyssavirus NLBat16<br>Rabies lyssavirus NLBat18<br>Rabies lyssavirus NLBat19<br>Rabies lyssavirus NLBat20<br>Rabies lyssavirus NLBat36<br>Rabies lyssavirus NLBat38<br>Rabies lyssavirus NLBat39<br>Rabies lyssavirus NLBat40<br>Rabies lyssavirus NLBat46<br>Rabies lyssavirus NLBat8
			Poland	European bat 1 lyssavirus Rv66
	Tissue	1987, 1989, 1999, 2000, 2009, 2011, 2013, 2014, 2015	Spain	European bat 1 lyssavirus R75<br>European bat 1 lyssavirus R76<br>European bat 1 lyssavirus 56R00<br>European bat 1 lyssavirus 69R00<br>European bat 1 lyssavirus 69R99<br>European bat 1 lyssavirus 80R99<br>European bat 1 lyssavirus 200928458<br>European bat 1 lyssavirus 201127004<br>European bat 1 lyssavirus 201149008<br>European bat 1 lyssavirus 2011riglos

				European bat 1 lyssavirus 201238163<br>European bat 1 lyssavirus 201325895<br>European bat 1 lyssavirus 201338163<br>European bat 1 lyssavirus 201422916<br>European bat 1 lyssavirus 201427094<br>European bat 1 lyssavirus 201539226<br>European bat 1 lyssavirus 201539228<br>European bat 1 lyssavirus 201544034<br>European bat 1 lyssavirus 201548093<br>European bat 1 lyssavirus RV119
	Tissue	1993, 2002	Switzerland	European bat 2 lyssavirus 02053SWI European bat 2 lyssavirus TW1392/93
	Tissue	2018	United Kingdom	European bat 1 lyssavirus EBLV-1b
