# Supplementary material for: Bat-Borne Viruses and Pandemic Risk: Could Europe Be an Emergence Hotspot?
Source: Viruses. 2026 May 2;18(5):535. doi: 10.3390/v18050535 (PMC13211416; doi:10.3390/v18050535)
Supplement: Supplementary file 1 [file viruses-18-00535-s001.zip › Skowron et al. - Table S3.pdf]

Supplementary Table S3. Cases of *Reoviridae* family viruses isolation from bats in Europe.

Bat species	Sample type	Collection year	Sampling country	Viruses
Eptesicusserotinus	Feces	2009, 2010	Slovenia	Mammalian orthoreovirus SI-MRV02 Mammalian orthoreovirus SI-MRV04
Hypsugo savii	Tissues	2019	Italy	Mammalian orthoreovirus 3 127044-18<br>Mammalian orthoreovirus 3 127044-19<br>Mammalian orthoreovirus 3 127044-23<br>Mammalian orthoreovirus 3 127044-38<br>Mammalian orthoreovirus 3 127044-47<br>Mammalian orthoreovirus 3 127044-68
Miniopterusschreibersii	Feces	2014	Serbia	Rotavirus J BO4351/Ms/2014
Myotisdasycneme	Feces	2016	Denmark	Rotavirus H RVH/18802-1/M.das/DK/2016
Myotisdaubentonii		2008	Germany	Bat rotavirus BatRV/SW78-39/Myo_dau/DEU/2008
	Feces	2012	Slovenia	Mammalianorthoreovirus SI-MRV03
	Feces, tissue	2019	Switzerland	Bat rotavirus BatRVH/Bat-wt/CH/Myo_dau/2019<br>Bat rotavirus BatRVH/Bat-wt/CH/Myo_dau2/2019<br>Bat rotavirus BatRVH/Bat-wt/CH/Myo_dau3/2019<br>Bat rotavirus BatRVH/Bat-wt/CHE/Myo_dau/2019<br>Bat rotavirus BatRVH/Bat-wt/CHE/Myo_dau2/2019<br>Bat rotavirus BatRVH/Bat-wt/CHE/Myo_dau3/2019
Myotisemarginatus	Feces	2009	Slovenia	Mammalianorthoreovirus SI-MRV06
Myotismyotis	Feces	2008	Slovenia	Mammalianorthoreovirus SI-MRV05
Myotismystacinus	Tissue	2009	France	Bat rotavirus b8
	Tissue	2009	Germany	Mammalian Orthoreovirus strain Bat/Germany/19/09<br>Mammalian Orthoreovirus strain Bat/Germany/21/09
Myotisnattereri	Feces	2018	Italy	Mammalian orthoreovirus MRV/18RS29002/M.nattereri/Italy/2018
Pipistrellus		2014	Netherlands	Rotavirus A RVA/Bat-wt/NDL/NPpipi1/2014/GxP[44]
Pipistrelluskuhlii	Feces, tissue, mix=feces and tissue	2011, 2012, 2019	Italy	Mammalian orthoreovirus 3 T3/Bat/Italy/130366/2011<br>Mammalian orthoreovirus 3 T3/Bat/Italy/155012/2011

Mammalian orthoreovirus 3 T3/Pipistrellus_khulii/Italy/130366/2011
Mammalian orthoreovirus 3 T3/Pipistrellus_khulii/Italy/155012/2011
Mammalian orthoreovirus 3 T3/Pipistrellus_khulii/Italy/206645-53/2011
Mammalian orthoreovirus 3 T3/Pipistrellus_khulii/Italy/206645-54/2011
Mammalian orthoreovirus 3 T3/Pipistrellus_khulii/Italy/206645-56/2011
Mammalian orthoreovirus 3 T3/Pipistrellus_khulii/Italy/206645-57/2011
Mammalian orthoreovirus 3 T3/Pipistrellus_khulii/Italy/206645-58/2011
Mammalian orthoreovirus 3 T3/Pipistrellus_khulii/Italy/206645-60/2011
Mammalian orthoreovirus 3 T3/Pipistrellus_Khulii/Italy/206645-63/2011
Mammalian orthoreovirus 3 T3/Pipistrellus_khulii/Italy/206645-64/2011
Mammalian orthoreovirus 3 T3/Pipistrellus_Khulii/Italy/5515-14/2012
Mammalian orthoreovirus 3 T3/Pipistrellus_Khulii/Italy/5515-2/2012
Mammalian orthoreovirus 3 T3/Pipistrellus_kuhlii/Italy/206645-53/2011
Mammalian orthoreovirus 3 T3/Pipistrellus_kuhlii/Italy/206645-54/2011
Mammalian orthoreovirus 3 T3/Pipistrellus_kuhlii/Italy/206645-56/2011
Mammalian orthoreovirus 3 T3/Pipistrellus_kuhlii/Italy/206645-57/2011
Mammalian orthoreovirus 3 T3/Pipistrellus_kuhlii/Italy/206645-58/2011
Mammalian orthoreovirus 3 T3/Pipistrellus_kuhlii/Italy/206645-60/2011
Mammalian orthoreovirus 3 T3/Pipistrellus_kuhlii/Italy/206645-63/2011
Mammalian orthoreovirus 3 T3/Pipistrellus_kuhlii/Italy/206645-64/2011
Mammalian orthoreovirus 3 T3/Pipistrellus_kuhlii/Italy/5515-1/2012
Mammalian orthoreovirus 3 T3/Pipistrellus_kuhlii/Italy/5515-14/2012
Mammalian orthoreovirus 3 T3/Pipistrellus_kuhlii/Italy/5515-2/2012
Mammalian orthoreovirus 3 T3/Pipistrellus_kuhlii/Italy/5515-3/2012
Mammalian orthoreovirus 3 T3/Pipistrellus_kuhlii/Italy/5515-4/2012
Mammalian orthoreovirus 3 127044-39

				Mammalian orthoreovirus 3 127044-50 Mammalian orthoreovirus 3 127044-51 Mammalian orthoreovirus 3 127044-53 Mammalian orthoreovirus 3 127044-75
	Mix=feces (living bats); brain, heart, lung, intestine, and spleen combined with liver (dead bats)	2019	Switzer-land	Bat rotavirus BatRVA/Bat-wt/CH/Pip_kuh/2019
Pipistrelluspipistrellus	Tissue	2019	Switzer-land	Bat rotavirus BatRVA/Bat-wt/CH/Pip_pip/2019 Bat rotavirus BatRVA/Bat-wt/CH/Pip_pip2/2019
Plecotusauritus	Tissue	2008	Germany	Mammalian Orthoreovirus strain T3/Bat/Germany/342/08
Pteropusvampyrus	Swabs	2010	Italy	PteropineorthoreovirusIndonesia/2010
Rhinolophus		2008	Bulgaria	Bat rotavirus BatRV/BB89-D/Rhi_spec/BGR/2008
Rhinolophusblasii		2008	Bulgaria	Bat rotavirus BatRV/BB89-15/Rhi_bla/BGR/2008 Rotavirus A RVA/Bat-wt/BGR/BB89-15/2008/G3P3
Rhinolophuseuryale		2008	Bulgaria	Bat rotavirusBatRV/BBR89-2/Rhi_eur/BGR/2008 Bat rotavirus-BatRV/BR89-60/Rhi_eur/BGR/2008 Rotavirus A RVA/Bat-wt/BGR/BR89-60/2008/G3P3
Rhinolophusferrumequinum	Feces	2019	Switzer-land	Bat rotavirus BatRVA/Bat-wt/CH/Rhi_fer/2019
Rhinolophushipposideros	Feces	2019	Switzer-land	Bat rotavirus BatRVA/Bat-wt/CH/Rhi_hip/2019
	Feces	2011	Italy	Mammalian orthoreovirus 3 T3/Rhinolophus_hipposideros/Italy/191797/2011
Tadaridateniotis	Tissue	2011	Italy	Mammalian orthoreovirus 3 T3/Tadarida_teniotis/Italy/206645-50/2011 Mammalian orthoreovirus 3 T3/Tadarida_teniotis/Italy/206645-51/2011
Vespertiliomurinus	Mix=feces and tissue	2011	Italy	Mammalian orthoreovirus 3 T3/Vespertilio_murinus/Italy/206645-31/2011 Mammalian orthoreovirus 3 T3/Vespertillio_murinus/Italy/206645-31/2011
unclassified Chiroptera	Tissue		Germany	Bat orthoreovirus T3/Bat/Germany/324/08 EuB-ReoV3 Common pipistrelle bat rotavirus EuB-ReoV2 Noctule bat orbivirus EuB-ReoV1
