# Supplementary material for: Bat-Borne Viruses and Pandemic Risk: Could Europe Be an Emergence Hotspot?
Source: Viruses. 2026 May 2;18(5):535. doi: 10.3390/v18050535 (PMC13211416; doi:10.3390/v18050535)
Supplement: Supplementary file 1 [file viruses-18-00535-s001.zip › Skowron et al. - Table S4.pdf]

Supplementary Table S4. Cases of *Calciviridae* family viruses isolation from bats in Europe.

Bat species	Sample type	Collection year	Sampling country	Viruses
Eptesicusserotinus	Feces	2013	Hungary	Bat calicivirus BtCalV/BS58/HUN/2013
Myotisalcathoe	Feces	2013	Hungary	CalicivirusBtCalV/EP38/HUN/2013
Myotisdaubentonii	Feces	2015, 2018	Denmark	Bat calicivirus BtCV/21164-6-A/M.dau/DK/2015<br>Bat calicivirus BtCV/21164-6-B/M.dau/DK/2015<br>Bat calicivirus BtCV/OV-157/M.dau/DK/2018
		2013	Hungary	Bat calicivirus BtCalV/M63/HUN/2013
